# *Adam19* Deficiency Impacts Pulmonary Function: Human GWAS Follow-up in a Mouse Knockout Model

**DOI:** 10.1007/s00408-024-00738-7

**Published:** 2024-08-17

**Authors:** Huiling Li, John S. House, Cody E. Nichols, Artiom Gruzdev, James M. Ward, Jian-Liang Li, Annah B. Wyss, Ezazul Haque, Matthew L. Edin, Susan A. Elmore, Beth W. Mahler, Laura M. Degraff, Min Shi, Darryl C. Zeldin, Stephanie J. London

**Affiliations:** 1 Immunity, Inflammation and Disease Laboratory, Division of Intramural Research, National Institute of Environmental Health Sciences, 111 TW Alexander Drive, MD A3-05, PO Box 12233, Research Triangle Park, North Carolina 27709 USA; 2 Biostatistics & Computational Biology Branch, Division of Intramural Research, National Institute of Environmental Health Sciences, Research Triangle Park, North Carolina USA; 3 Whitsell Innovations, Inc., Chapel Hill, North Carolina USA; 4 Reproductive & Developmental Biology Laboratory, Division of Intramural Research, National Institute of Environmental Health Sciences, Research Triangle Park, North Carolina USA; 5 Integrative Bioinformatics Support Group, Division of Intramural Research, National Institute of Environmental Health Sciences, Research Triangle Park, North Carolina USA; 6 Cardiovascular Institute, Beth Israel Deaconess Medical Center, Boston, Massachusetts USA; 7 Cellular & Molecular Pathology Branch, Division of the National Toxicology Program, National Institute of Environmental Health Sciences, Research Triangle Park, North Carolina USA

**Keywords:** Meltrin beta, RNA-Seq, Lung function, FlexiVent, Spirometry, Inflammation

## Abstract

**Purpose:**

Over 550 loci have been associated with human pulmonary function in genome-wide association studies (GWAS); however, the causal role of most remains uncertain. Single nucleotide polymorphisms in a disintegrin and metalloprotease domain 19 (*ADAM19*) are consistently related to pulmonary function in GWAS. Thus, we used a mouse model to investigate the causal link between *Adam19* and pulmonary function.

**Methods:**

We created an *Adam19* knockout (KO) mouse model and validated the gene targeting using RNA-Seq and RT-qPCR. Mouse body composition was assessed using dual-energy X-ray absorptiometry. Mouse lung function was measured using flexiVent.

**Results:**

Contrary to prior publications, the KO was not neonatal lethal. KO mice had lower body weight and shorter tibial length than wild-type (WT) mice. Their body composition revealed lower soft weight, fat weight, and bone mineral content. *Adam19* KO had decreased baseline respiratory system elastance, minute work of breathing, tissue damping, tissue elastance, and forced expiratory flow at 50% forced vital capacity but higher FEV_0.1_ and FVC. *Adam19* KO had attenuated tissue damping and tissue elastance in response to methacholine following LPS exposure. *Adam19* KO also exhibited attenuated neutrophil extravasation into the airway after LPS administration compared to WT. RNA-Seq analysis of KO and WT lungs identified several differentially expressed genes (*Cd300lg, Kpna2, and Pttg1*) implicated in lung biology and pathogenesis. Gene set enrichment analysis identified negative enrichment for TNF pathways.

**Conclusion:**

Our murine findings support a causal role of *ADAM19*, implicated in human GWAS, in regulating pulmonary function.

**Supplementary Information:**

The online version contains supplementary material available at 10.1007/s00408-024-00738-7.

## Introduction

Spirometric measures of lung function are routinely used in clinical medicine to diagnose chronic obstructive pulmonary disease (COPD) and monitor its severity along with asthma and other lung diseases. Lower function is related to mortality independently of other risk factors [1]. Human genome-wide association studies (GWAS) have identified genetic variants in over 550 genes related to pulmonary function [2]. Among these, variants in a disintegrin and metalloproteinase domain 19 (*ADAM19*) have been consistently associated with forced expiratory volume in the first second (FEV_1_) [3, 4], the ratio of FEV_1_ to forced vital capacity (FVC) [1–3, 5–8], peak expiratory flow (PEF) [3, 4], and COPD [9, 10]. However, while GWAS is powerful for identifying genetic associations, it cannot assign causality. Therefore, we followed up on these human GWAS findings using mouse models.

ADAM19 protein is primarily membrane-bound in various tissues and is expressed in the lung [11, 12]. It functions by shedding proteins, such as tumor necrosis factor (TNF), from the cell membrane by activating the catalytic site in its exon 11 [13–15]. Shed proteins can trigger signal transduction and regulate inflammation and other pathological processes [16–18].

The original study of genetic disruption of *Adam19* in mice showed it to be essential for cardiac development [19]; mice deficient in *Adam19* exons 10–12 exhibited severe cardiac defects, with only 5% surviving to postnatal day 8. Therefore, expecting early lethality and lack of specific ADAM19 antibodies, we created a reporter mouse by replacing exons 6 and 7 in *Adam19* with a tdTomato red gene construct. We expected the heterozygous reporter mouse to be viable and thus usable to visualize the tissue distribution of ADAM19 and study the role of *Adam19* in organogenesis, especially of the lungs. Mice with homozygous *Adam19-tdTomato* alleles are equivalent to *Adam19* knockout (KO); surprisingly, they were viable. Thus, we performed RNA sequencing (RNA-Seq) and reverse transcription-quantitative polymerase chain reaction (RT-qPCR) to validate the knockout of *Adam19*. We confirmed the knockout and measured pulmonary function in adult *Adam19* KO mice and WT controls.

A role for *Adam19* in lung function was identified for the first time in 2009 [5]. The mechanistic understanding of this association is not yet well developed. The extracellular matrix influences lung mechanical properties and, thus, lung function parameters [20, 21]. *Adam19* increases extracellular matrix deposition in response to TGF beta, which is important in many lung diseases [22]. These suggest the mechanistic link between *Adam19* and lung function. *Adam19* promotes the release of TNF, which affects lung function and contributes to lung diseases such as asthma, COPD, and lung fibrosis [23, 24]. Therefore, we examined whether genetic deficiency of *Adam19* affects airway responsiveness and the immune cell profile of bronchoalveolar lavage fluid (BALF) following the administration of LPS, a classical inflammatory stimulant. *Adam19* has been shown to influence osteoblast differentiation and thus affect bone formation [25]. Due to our knockout’s smaller appearance, we also measured body weight, tibial length, and body composition. Some results were previously presented as an abstract.[26].

## Materials and Methods

Detailed methods are in the online supplementary information.

### *Adam19* Gene Targeting Scheme and Murine Studies

**Fig. 1:**
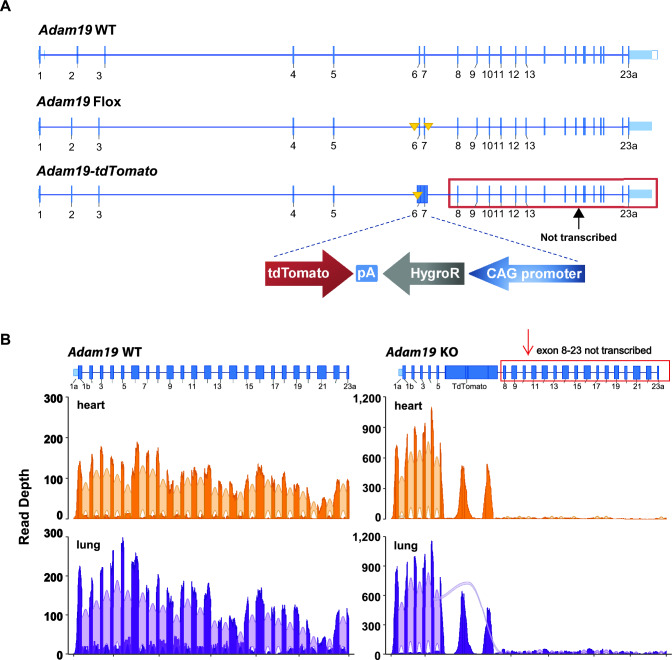
Gene targeting scheme (**A**) for *Adam19-tdTomato* allele and validation by RNA-Seq (**B**). A. *Adam19* WT: Endogenous wild-type locus. *Adam19* Flox: *Adam19* conditional null (“flox”) allele with exon 6 and 7 floxed by LoxP sites (solid yellow triangles). Please see the supplemental methods for a detailed description of the floxed allele. *Adam19-tdTomato*: *Adam19* mutant allele in which exons 6 and 7 are replaced by the tdTomato construct, disrupting *Adam19* gene expression. Homozygosity for *Adam19-tdTomato* alleles is equivalent to *Adam19* knockout (KO). Each blue box represents an exon; the exon number is underneath. pA: polyA; hygroR: Hygromycin Resistance. CAG:CMV enhancer, chicken beta-Actin promoter, and rabbit beta-Globin splice acceptor site. B. Read densities of *Adam19* exons and junctions in WT and KO mice by RNA-Seq analysis. The blue boxes represent exons. In the *Adam19* KO, exons 6 and 7 were replaced by the tdTomato construct and showed minimal transcript expression from exon 8 through the end of the *Adam19* gene. Bold orange or bold purple regions represent aggregate sequence read depth across the *Adam19* WT (left panel) or KO (right panel) gene locus. Light orange or light purple arcs indicate sequence reads whose alignments represent observed splice junctions. The arc width indicates the aggregate number of junction reads. No in-frame splicing events were detected from exon 5 to any downstream *Adam19* exons. *n* = 3 mice per genotype per heart or lung tissue

*Adam19* exons 6 and 7 were replaced with an in-frame tdTomato construct. Homozygosity for *Adam19-tdTomato* alleles is equivalent to the *Adam19* KO (Fig. 1A).

Mice in this study were all males aged 9–13 week with 129S6/SvEvTac background, confirmed by MiniMUGA genome genotyping arrays [27]. The use of animals followed NIH guidelines and was approved by the NIEHS Animal Care and Use Committee.

### RNA-Seq and RT-qPCR

Strand-specific RNA-Seq was conducted on Illumina NextSeq. Sequence coverage was visualized with sashimi plots [28]. The absence of *Adam19* transcript in KO was validated by both RNA-Seq and RT-qPCR.

### Differential Gene Expression and Gene Set Enrichment Analysis

RNA transcript reads were quantified versus GENCODE vM33 comprehensive transcripts (mm39) using Salmon 1.10.0 [29]. Differential gene expression was performed by limma-voom 3.54.2 [30], using thresholds for false discovery rate (FDR)-adjusted *p* < 0.05, fold change ≥ 1.5, and a group mean normalized transcript abundance ≥ 6 in at least one sample group.

We used gene set enrichment analysis (GSEA) [31] to identify significantly enriched pathways (FDR *p* < 0.25) for genes differentially expressed between *Adam19* WT and KO by RNA-Seq. GSEA was conducted using the Broad Molecular Signature Database (MSigDB, v2023.2.Mm) hallmark gene sets collection.

### Embryo Organogenesis

E18.5 embryos were sectioned sagittally (5 µm thickness) and stained with hematoxylin and eosin. A pathologist (SAE) evaluated the tissue sections, focusing on the cellular structure of the heart, lung, valves (heart, aortic, and pulmonary), adrenal glands, and diaphragm.

### Assessment of Body Weight, Tibia Length, and Body Composition

Body weight was measured using a top-loading scale with an accuracy of 0.01 g. Tibia length was measured with a ruler with an accuracy of 0.1 cm. Body composition parameters [32] were assessed using the Faxitron Dual-energy X-ray Absorptiometry (DXA) imaging system.

### Pulmonary Function Parameter Measurements

Pulmonary function parameters were measured using flexiVent FX2 with a negative pressure-driven forced expiratory extension. Mouse body weight was entered at the time of lung function determination to adjust the perturbation amplitude. Baseline measurements and responses to methacholine doses were assessed [33, 34]. For LPS experiments, mice received LPS or saline via oropharyngeal aspiration (OPA). After four hours, lung function parameters were measured using the same flexiVent procedure.

### Lung Histology Examination

To better delineate the morphologic impacts of *Adam19* perturbation, we examined collagen content using Masson’s trichrome (MT) and extracellular matrix accumulation using periodic acid-Schiff (PAS) staining in lung tissue sections from naïve mice.

### Immune Cell Profile and Cytokine Analysis in BALF in LPS Exposed Mice

BALF was collected from each mouse by rinsing the airways [33]. We counted cells using an automated cell counter and determined the percentage of different immune cell types from cytospin slides. For LPS experiments, mice received LPS or saline via OPA and were euthanized after 4 h. BALF was then collected and analyzed as described above. Cytokine concentrations (IL-1b, IL-2, IL-6, KC, MCP-1, MIP-1a, MIP-1b, and TNF-a) were determined using a custom Bio-Plex Pro Mouse Cytokine 8-plex assay.

### Statistical Analyses

We conducted linear regression analyses to assess genotype differences in body weight, body composition parameters, and tibia length. Genotype differences in lung function parameters were analyzed at baseline using a general linear model. The maximum response to methacholine doses (normalized to the response to PBS aerosol) was analyzed using a linear mixed-effect model with a random intercept. Genotype differences in dose–response slopes in response to LPS were assessed. All analyses of lung function parameters were adjusted for body weight. Changes in cell counts of each immune cell type (except eosinophils present in just one mouse) across genotypes following LPS exposure were analyzed using linear regression. Linear regression with a robust sandwich estimator was used for cytokine data analysis. We used R version 4.2.2 for analyses and plots.

## Results

### Gene Targeting Scheme and Validation for *Adam19* KO Mouse

We replaced the *Adam19* exons 6 and 7 with the dTomato red gene open reading frame and anti-sense Hygromycin resistance (HygroR) cassette (Fig. 1A). RNA-Seq confirmed the absence of *Adam19* transcript encoding the active catalytic site of the functional protein. In both heart and lung, *Adam19* mRNA expression was minimal from exon 8 through 23 in KO mice (Fig. 1B and S1). Small amounts of exon 5 to 8 splicing transcripts were observed in lungs but not hearts in KO mice (Fig. 1B).

The detailed gene transcript structure in the heart and lung (WT and KO), derived from RNA-Seq analysis, is shown in Fig. S1 (available via the NIEHS-hosted track hub in the UCSC Genome Browser using the URL https://genome.ucsc.edu/cgi-bin/hgTracks?genome=mm39_adam19tdt&hubUrl=https://orio.niehs.nih.gov/ucscview/Adam19/hub.txt. The RNA-Seq data discussed here were deposited in NCBI’s Gene Expression Omnibus [35] under the accession number GSE183318 (https://www.ncbi.nlm.nih.gov/geo/query/acc.cgi?acc=GSE183318).

Follow-up analysis with RT-qPCR (TaqMan and SYBR Green assays) confirmed the absence of *Adam19* mRNA expression from exon 6 through 23 in both heart and lung in KO mice (Fig. S2), additionally validating the absence of exon 11, which encodes the active catalytic site of the ADAM19 protein [19].

Because sufficiently specific ADAM19 antibodies were unavailable, we created an *Adam19-tdTomato* reporter mouse model to visualize the tissue distribution of ADAM19. However, we could not detect fluorescence from the ADAM19-tdTOMATO fusion protein, possibly due to tdTOMATO protein misfolding. Because of nonspecific staining, we cannot be certain of the detection of tdTOMATO protein in the heterozygous *Adam19-tdTomato* mouse lung tissue (Figure S3). The *Adam19* transcript from the KO contains only the first five exons, representing only 12% (333 nucleotides) of the full-length *Adam19* open reading frame (2760 nucleotides).

### Differential Gene Expression and Gene Set Enrichment Analysis

Differential gene expression analysis of 3 WT and 3 KO mice revealed few statistically significant changes in gene expression patterns in the lungs of KO compared to WT mice. We observed increased expression of pituitary tumor-transforming gene 1 (*Pttg1*), karyopherin subunit alpha 2 (*Kpna2*), and CD300 molecule like family member g (*Cd300lg*) in KO lungs. (Table 1, Fig. S4). Other genes with increased expression in the KO include *Rpl14-ps1*, *Gm21970*, and *Gm11131*. (Table 1, Fig. S4). KO lungs had decreased *AA465934*, *Gm18860*, *Gm12663*, and *Gm10184*. Consistent with expectation, the WT mice did not exhibit the *Adam19-tdTomato* fusion gene expression.

**Table 1:** Differentially Expressed Genes in *Adam19* KO versus WT Mouse Lungs Based on RNA^a^

Gene Name	Chr	Gene Start (bp)	Gene End (bp)	Fold Change	Adjusted *p*-Value	MGM
*Rpl14-ps1*	chr7	44,974,389	44,975041	797.9	0.0247	10.64
*Gm21970*	chr16	91,180,749	91,222,722	396.4	0.0162	9.63
*Gm11131*	chr17	35,595,997	35,599,268	33.0	0.0138	6.04
*Pttg1*	chr11	43,311,077	43,317,078	16.3	0.0006	9.32
*Kpna2*	chr11	106,879,455	106,890,367	8.8	0.0291	9.10
*Cd300lg*	chr11	101,932,335	101,946,446	5.4	0.0474	7.47
*AA465934*	chr11	83,182,513	83,185,527	− 15.1	0.0044	6.59
*Gm18860*	chr8	72,156,849	72,157,612	− 32.1	0.0474	6.01
*Gm12663*	chr11	16,585,109	16,586,066	− 55.2	0.0007	6.53
*Gm10184*	chr17	90,215,890	90,217,877	− 426.8	0.0138	9.74

*Chr* Chromosome; *bp* base pair; *MGM* maximum group mean, the highest normalized group mean abundance for each gene

^a^Considered statistically significant with false discovery rate adjusted *p* < 0.05, fold change magnitude ≥ 1.5 (positive fold change means increase, and negative fold change means decrease), and MGM ≥ 6. The table was sorted by fold change descending; *n* = 3 per genotype. The *Adam19* WT and *Adam19-tdTomato* loci were masked from the differentially expressed gene analysis because the two loci only exist in their respective genotypes

Gene Set Enrichment Analysis (GSEA) revealed that Myc targets, oxidative phosphorylation, E2F targets, unfolded protein response, protein secretion, TNF alpha signaling via NFkB, G2M checkpoint, and DNA repair gene sets were negatively correlated with *Adam19* KO (Table S1). In contrast, the mitotic spindle gene set was positively correlated with *Adam19* KO (Table S1).

### Embryo Organogenesis

A pathologist evaluated three E18.5 KO embryos and two E18.5 WT littermates for tissue or organ abnormalities and did not identify lesions associated with tetralogy of Fallot in the heart (overriding aorta, pulmonic stenosis, ventricular septal defect, and right ventricular hypertrophy) as previously reported [19, 36] nor abnormalities in the lungs, diaphragms, and adrenal glands (Fig. S5).

### Reduced Body Weight, Shorter Tibial Length, and Altered Body Composition in *Adam19* KO Mice

**Fig. 2:**
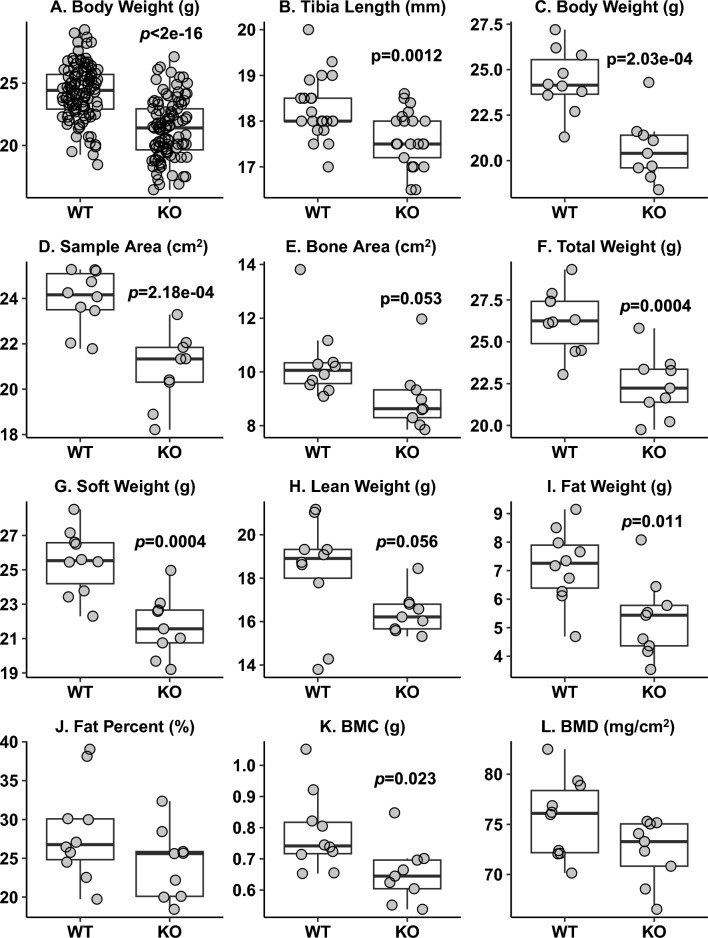
*Adam19* KO mice have reduced body weight, shorter tibia length, and altered body composition. A. Body weight measured using a top-loading scale (WT: *n* = 114, KO: *n* = 104). B. Tibia length measured using a ruler (WT: *n* = 24, KO: *n* = 20). C: Body weight of mice for measuring body composition. D-L: Body composition parameters obtained using dual-energy X-ray absorptiometry (WT: *n* = 10, KO: *n* = 9). Total weight = soft weight + bone mineral content (BMC), soft weight = lean weight + fat weight, Fat % = fat weight/soft weight in percentage, BMD = bone mineral density = BMC/bone area. *p* values < 0.05 for differences in the parameter by genotype are displayed (**A**–**L**)

We weighed 114 WT and 104 KO mice at 9–13 week. Age-adjusted body weight (Fig. 2A) was significantly lower in *Adam19* KO than in WT mice. WT mice continued gaining weight through the assessment period, while the KO stopped gaining weight at nine weeks (Fig. S6). Tibia length was shorter in KO than in WT (20 KO, 24 WT) (Fig. 2B). Using DXA, we measured body composition on 10 KO and 10 WT mice 9.9 to 13.1 week and subsequently excluded one outlier KO. In addition to lower body weight (Fig. 2C), compared to WT, KO had reduced sample area (Fig. 2D), bone area (Fig. 2E), total weight (Fig. 2F), soft weight (Fig. 2G), lean weight (Fig. 2H), fat weight (Fig. 2I), and bone mineral content (BMC) (Fig. 2K). There were no significant differences in %fat (Fig. 2J) or bone mineral density (BMD) (Fig. 2L). Values used in Fig. 2 are shown in Table S2.

### Pulmonary Function Parameters Altered in *Adam19* Deficient Naïve Mice

**Fig. 3:**
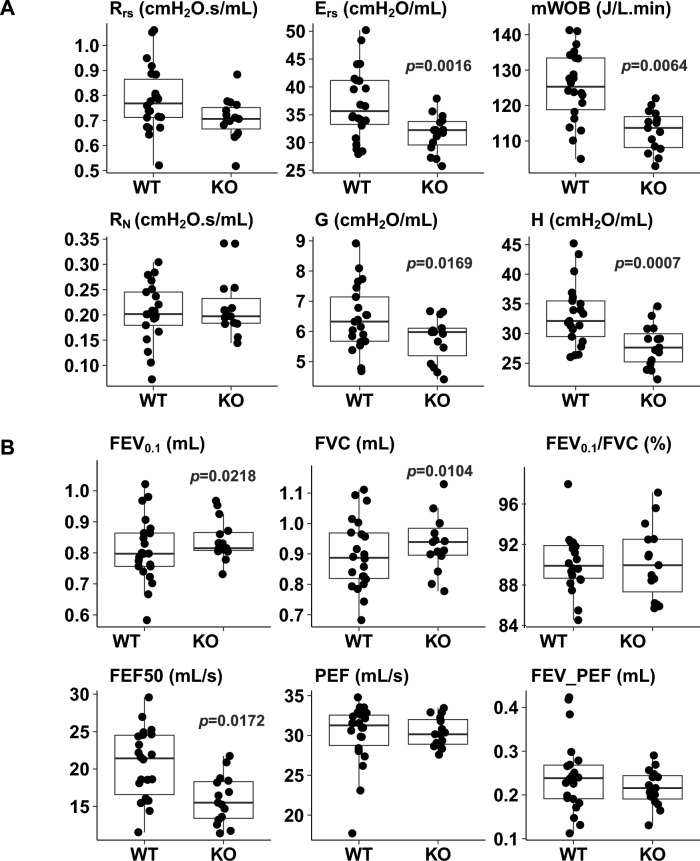
*Adam19* deficiency alters (**A**) baseline mechanics and (**B**) spirometry parameters determined by flexiVent. *n* = 22 for WT, *n* = 15 for KO. R_rs_ = resistance of the respiratory system; E_rs_ = elastance of the respiratory system; mWOB = minute work of breathing (the work required to breath-in on a minute basis); J = joule (one joule is the work required to move 1 L of gas through a 10-cmH_2_O pressure gradient). R_N_ = Newtonian resistance; G = tissue damping; H = tissue elastance; FEV_0.1_ = forced expiratory volume in 0.1 s; FVC = forced vital capacity; FEV_0.1_/FVC = the ratio of FEV_0.1_ over FVC in %; FEF50 = Forced expiratory flow at 50% FVC; PEF = Peak expiratory flow; FEV_PEF = Forced expiratory volume at peak expiratory flow. *p* < 0.05 for differences in the parameter by genotype are displayed

We measured baseline lung function in 37 mice (22 WT and 15 KO). *Adam19* KO mice exhibited reduced elastance of the respiratory system (E_rs_), minute work of breathing (mWOB), tissue damping (G), and tissue elastance (H) (Fig. 3A). Additionally, forced expired flow at 50% FVC (FEF50) was lower in the KO compared to WT mice (Fig. 3B). However, KO had higher FEV_0.1_ and FVC (Fig. 3B). No genotype differences were observed in resistance of the respiratory system (R_rs_) or Newtonian resistance (R_N_) (Fig. 3A) nor FEV_0.1_/FVC, PEF or FEV_ PEF (Fig. 3B) nor in airway responsiveness to methacholine (Fig. S7). Values used in Fig. 3 are shown in Table S3.

### Airway Responsiveness to Methacholine Attenuated in *Adam19* Deficient Mice Exposed to LPS

**Fig. 4:**
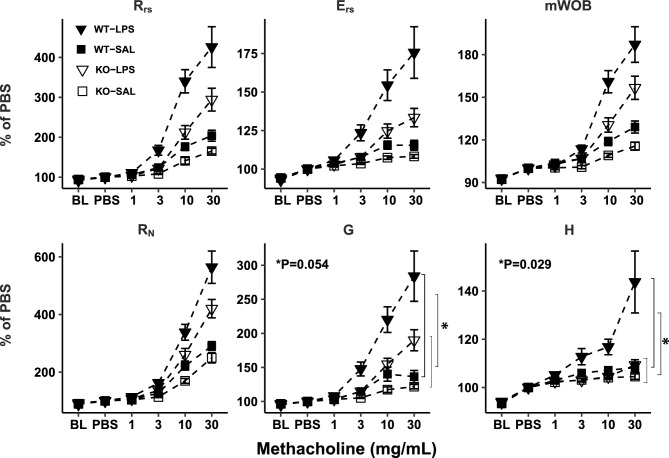
*Adam19* deficient mice have reduced airway responsiveness to methacholine following LPS exposure. The maximum response to methacholine at each dose was expressed as a percentage of the maximum response at PBS. Means and standard errors of means are indicated as bar lines. R_rs_ = resistance of the respiratory system; E_rs_ = elastance of the respiratory system; mWOB = minute work of breathing; R_N_ = Newtonian resistance; G = tissue damping; H = tissue elastance; BL = baseline; PBS = phosphate buffered saline. Only the log methacholine doses (1, 3, 10, and 30 mg/ml) were used in the linear regression analysis. The responses normalized to PBS (PBS as 100%) were linear to log (methacholine doses). *p values ≤ 0.05 are shown for the genotype difference of the slope difference of the response to methacholine following LPS exposure (vs. saline). *n* = 13 for WT-Saline, *n* = 20 for WT-LPS, *n* = 14 for KO-Saline, *n* = 21 for KO-LPS

*Adam19* has been shown to promote inflammation [17, 18, 37]. Therefore, we assessed genotype differences in airway responsiveness induced by LPS. We did not detect differences in LPS response (vs. saline) across genotypes for baseline lung function parameters (Fig. S8). However, LPS-induced differences (vs. saline) in the slope estimates for the methacholine dose–response curves were lower in KO than WT for tissue damping (G) and tissue elastance (H) (Fig. 4).

### Lung Histology Examination

We did not find genotype differences in Masson’s trichrome and Periodic Acid-Schiff (PAS) analyses in mouse lung tissue (Fig. S9).

### Immune Cell Differential Analysis in BALF in Mice Following LPS Exposure

**Fig. 5:**
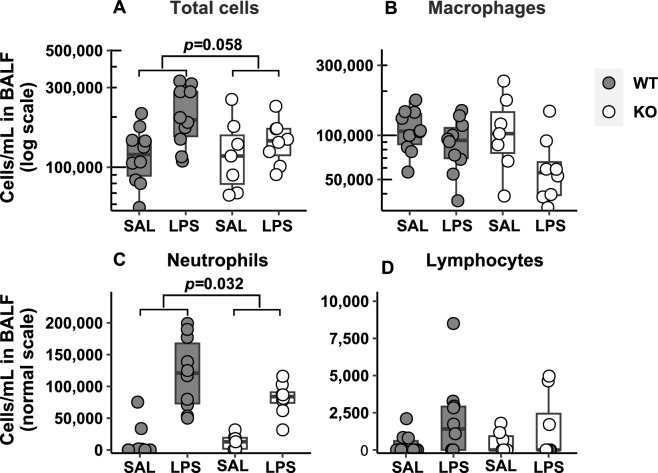
Analysis of differential immune cell counts in bronchoalveolar lavage fluid (BALF) in mice following LPS exposure. Increases in neutrophil number (C) induced by LPS (vs. saline) were lower in *Adam19* KO than in WT mice (46% fewer cells, *p* = 0.032). The degree of increase in total cells (A) following LPS (vs. saline) was lower in KO (the ratio of the LPS effect between the *KO* and *WT* = 0.61, 95% confidence interval (*CI*) = 0.36–1.02, *p* = 0.058). No genotype differences of LPS effects were identified for macrophages (B) and lymphocytes (D). SAL = saline, WT: *n* = 10 (SAL), 10 (LPS); KO: *n* = 7 (SAL), 8 (LPS)

As expected, counts of total cells and neutrophils increased in WT and KO mice following LPS exposure. However, the degree of increase in neutrophil counts was lower in KO than in WT (46% fewer cells, *p* = 0.032, Fig. 5C). Similarly, the increase in total cells was lower in the KO compared to WT (Fig. 5A). These results suggest that the *Adam19* KO mice show reduced responsiveness to LPS compared to WT regarding immune cell profiles. There were no genotype differences in LPS-induced changes in macrophage or lymphocyte counts (Fig. 5B, 5D).

### Cytokine Analysis in BALF in LPS Exposed Mice

As expected, we observed increases in several inflammatory cytokines in response to LPS vs. saline (IL-6, KC, MCP-1, MIP-1a, MIP-1b, and TNF-a); these did not differ by genotype (Fig. S10). IL-1b and IL-2 levels in all mice were below the detection range (not shown).

## Discussion

*Adam19* has been consistently associated with pulmonary function in human GWAS. However, GWAS alone cannot establish causality. Mouse models are useful in investigating the causal role of loci identified in GWAS in pulmonary function. We successfully generated a novel *Adam19* knockout mouse model and confirmed gene disruption through RNA-Seq and RT-qPCR analysis. Contrary to previous publications, our KO mice are viable and generally healthy, without the lethal cardiac abnormalities reported previously [19, 36].

We considered factors potentially contributing to the discrepancy in the viability of our KO compared to prior work [19, 36]. Firstly, methods for producing the knockout differed between studies. Kurohara et al*.* [19] replaced exons 10 through 12 with an antisense Neomycin resistance cassette, and Zhou et al. [36] introduced a gene trap 3’ of exon 14. We replaced exons 6 and 7 with an in-frame tdTomato construct.

Second, genetic disruption of a multi-exon locus, like *Adam19,* may generate novel transcript variants through alternative splicing; some may result in a neomorph that rescues ADAM19 deficiency. In contrast, others may be potentially toxic in the absence of ADAM19. Our RNA-Seq analysis was designed to detect alternative splice variants but identified no alternative *Adam19* splice variants and no active transcription from exons 8 through 23. Interestingly, we detected a novel splice variant in the lung, splicing from exon 5 to exon 8. However, this transcript led to a near-immediate nonsense mutation. Thirdly, the remaining gene structure in a knockout might influence its interaction with other genes or proteins, leading to different functional consequences. Our knockout mice expressed only the first five exons of *Adam19*, which do not include the sequences that encode the active catalytic sites of ADAM19. Transcriptions of exons 8 through 23 were nearly absent, providing confidence that no functional ADAM19 metalloproteinase domains were formed. While both genetic constructs in previous studies [19, 36] disrupt metalloprotease function, they may have generated truncated ADAM19 proteins that could interact with other proteins in a non-productive or dominant-negative manner.

Additional explanations for differences in the viability of the KO across studies include differences in the genetic background of the mouse lines used. Our *Adam19*-deficient allele was generated in 129S ES cells and subsequently maintained on the 129S6/SvEvTac background, whereas the other studies used mice with a mixed genetic background of 129 Sv and C57BL6/J [19, 36]. Further, in prior work, variability was seen in the penetrance of the observed cardiac phenotypes. In Kurohara’s ADAM19-deficient line, some mice survived to adulthood without severe cardiac defects besides enlarged hearts [19]. Partial penetrance of lethal phenotypes is common, so this phenotype variability is not surprising. However, it does suggest that the genetic disruption of *Adam19* is more complex than initially envisioned during our gene targeting design.

We do not know why our *Adam19*-deficient mice were viable without the noticeable cardiac defects observed previously [19, 36]. However, all available evidence and data strongly indicate that we had a functional knockout of the canonical ADAM19 protein despite the small sample size as a limitation. Moreover, our knockout had normal-appearing hearts, and there were no obvious survival disadvantages. Our knockout mice unlikely retained ADAM19 activity, given that exons 1 to 5 only encode for the first 111 of 920 amino acids of canonical ADAM19 protein but none of the active sites of metalloproteinase domains. In addition, *Adam19*’s first five exons appeared to have higher expression in heart and lung samples in KO than wildtype, possibly caused by either a feedback mechanism attempting to compensate for the functional loss of *Adam19* or by the absence of appropriate 3′ UTR elements for the consistent transcript turnover in mutant samples or both.

Our *Adam19* KO animals exhibited several notable phenotypic differences compared to their WT littermates, including reduced body weight, decreased tibia length, and altered body composition. Inoue et al. reported that *Adam19* was involved in osteoblast differentiation in mice [38], which may help explain why our *Adam19* knockouts have shorter tibias. Weerasekera et al. demonstrated a correlation between high ADAM19 expression in human peripheral blood mononuclear cells and BMI, relative fat, and TNF levels [37]. They also observed increased *Adam19* mRNA and ADAM19 protein in the liver tissue of mice fed a high-fat diet (HFD). In contrast, neutralizing ADAM19 protein with its antibody resulted in weight loss, reduced white fat accumulation, and decreased TNF protein levels in the liver of HFD-fed mice. These published findings provide insights into our observations of smaller body sizes, reduced body weight, and altered body compositions. Further, differentially expressed genes we identified included *Kpna2,* which was associated with body weight and BMI in human GWAS [39], and *Cd300lg,* which has been associated with increased intramyocellular lipid content and reduced fasting forearm glucose uptake in humans [40]. Additionally, GSEA enrichment in multiple pathways related to cell proliferation and metabolism could contribute to the anthropometric phenotype in our KO. Collectively, our data support the role of ADAM19 in regulating growth and body weight development.

Human GWAS have identified hundreds of variants in or near *ADAM19* that are significantly associated with lung function [2, 3, 5, 7, 10, 41]. In particular, the minor alleles of sentinel SNPs have been associated with lower FEV_1_/FVC and FEV_1_, including rs2277027 [5], rs11134789 [3, 10], and rs4331881 [2], which are in high linkage disequilibrium. However, other genome-wide significant variants displayed opposite effects with the minor allele associated with higher FEV_1_/FVC and FEV_1_, including rs1990950 and rs59327154. Several variants in *ADAM19* have also been associated with COPD, including rs2277027, rs1422795, rs11744671, and rs113897301, for which the minor allele was associated with an increased risk of COPD [9, 42, 43]. When we queried the Genotype-Tissue Expression (GTEx) Portal for gene expression, we noted hundreds of variants in *ADAM19* that implicate significant eQTLs in lung tissue, including sentinel SNPs rs11134789 and rs2277027, which were among the top most significant eQTLs and had minor alleles associated with increased expression [44]. Yet, other variants that had significant positive associations in GWAS had minor alleles associated with decreased expression. In a study combining UK Biobank GWAS data with gene expression data, protein level data and functional annotation, *ADAM19* met the criteria as a putative causal gene for FEV_1_/FVC as well as FEV_1_ and peak expiratory flow [3]. However, given the large number of *ADAM19* variants associated with lung function and COPD, as well as gene expression and the range of effects depending on the individual SNPs, it is difficult to pinpoint a single causal variant and, therefore, challenging to comment with certainty on the overall direction of effect. This is a known limitation of GWAS and highlights the importance of follow-up research utilizing fine-mapping and multi-omics data [45, 46] as well as mouse model approaches.

Critical to comparison with human GWAS, the *Adam19* KO mice also displayed altered baseline pulmonary function parameters, namely decreased elastance of the respiratory system, minute of work of breathing, tissue damping, tissue elastance, and declined forced expiratory flow at 50% forced vital capacity, as well as increased FEV_0.1_ and FVC. Because of the smaller size of our KO, we adjusted all statistical analyses of lung function parameters for weight to ensure the observed lung function differences by genotype were not due to the smaller size. Using flexiVent, lung function parameters were determined based on lung responses to frequency-dependent input signals. For input signals at a fixed breathing frequency, lung function parameters (Rrs, Ers) were captured. The Rrs and Ers reflect resistance and distensibility of the whole respiratory system, including airways, lung tissues, and chest wall [47]. For input signals at various frequencies, lung function parameters can be partitioned to reflect the contribution of different lung regions [48]. For example, Rn reflects the resistance of central airways. Tissue damping (G) closely relates to the resistance of peripheral lung tissue. Tissue elastance (H) reflects the elastic recoil of lung tissue. FEV_0.1_ simulates human FEV_1_ and FEV_0.1_/FVC simulates human FEV_1_/FVC. FVC is relevant to FVC in humans. In our knockout, we found consistent results for FEV_0.1_ and FVC. Perhaps, not surprisingly, we did not find a significant difference when taking the ratio. FEV_0.1_ was higher, while FEF50 was lower in our KO compared to WT; we would not necessarily expect directions of effect to be the same because the two parameters were uncorrelated in our data. We note that Kwon et al. reported mild COPD patients with normal FEV_1_ had reduced FEF25%-75%, which is equivalent to FEF50 in our study [49]. mWOB is correlated with airway compliance and was reduced in an emphysema mouse model and increased in a fibrosis mouse model [50]. Our data provide compelling evidence for a causal role of *ADAM19* in pulmonary function, confirming findings from human GWAS.

Collagen is the main constituent of lung connective tissues, which provides support in the bronchi, interstitium, and alveolar wall structures and plays an essential role in lung mechanics [51]. We did not observe any genotype differences between WT and KO for either lung histology (Fig. S5) in general or for collagen deposition (Fig. S9). This is consistent with our lung function findings—no change in the naïve KO for the baseline respiratory system resistance (Rrs) and Newtonian resistance (RN, reflecting conducting airway resistance) (Fig. 4) and the airway responsiveness to methacholine (Fig. S7). Of note, lung function differences were generally subtle, and thus, the lack of histologic differences is perhaps not surprising. As a limitation, our study did not investigate the roles of other connective tissue components in lung function.

The precise molecular mechanisms underlying these observations for lung function remain unknown. ADAM19 cleaves NEUREGULIN-1 (NRG1), an erythroblastic leukemia viral oncogene homolog (ERBB) receptor tyrosine kinases ligand. ERBB receptor ligands NRG1 and epidermal growth factor affect fetal surfactant synthesis in the developing mouse lungs [52]. ADAM19 has also been implicated in non-proteolytic functions, such as regulating neuromuscular junctions in murine embryos through Eph family receptor-interacting proteins (EPHRIN)-A5/EPHRIN-A4 signaling [53]. In addition, the cytoplasmic tail of ADAM19 has several Src homology 3 (SH3) binding sites that regulate protein–protein interactions. ADAM19 binds strongly to the scaffolding protein tyrosine kinase substrate with five SH3 domains and the Src tyrosine kinase, potentially influencing cytoskeletal functions that impact cell motility, contractility or tissue development [54]. Therefore, disruption of ADAM19 may have important effects on lung development, neuromuscular functions, tissue elastance, contractility, or other unidentified signaling processes.

Our differential gene expression analysis identified genes related to lung physiology and pathology. For example, increased *Kpna2* expression may contribute to altered lung function, consistent with publications that *KPNA2* genetic variation is associated with FEV_1_/FVC in human GWAS [2] and plays a role in lung cancer [55]. Our KO had increased Pttg1 expression. *Pttg1* is involved in cell cycle regulation [56] and the development of lung cancer [57], suggesting its role in the lungs. Interestingly, some of these differentially expressed genes were on chromosome 11, where *Adam19* is located; this might imply additional effects of the KO construct. Chromosome 11 has a high gene density; the genes we detected on chromosome 11 were proportional to the number of genes on other chromosomes. The gene expression differences were relatively small between our *Adam19* WT and KO. We confirmed 1) the absence of *Adam19* transcription and 2) the absence of novel *Adam19* splicing variants that might rescue the lethal cardiac phenotype as expected based on the literature, which were our primary goals of the RNA-Seq analyses. The small sample size was a limitation to identify the differentially expressed genes across the genome definitively. We identified a limited set of differentially expressed genes with this modest sample size.

Given our observation of reduced neutrophil infiltration in BALF following LPS exposure in the *Adam19* knockout, we investigated whether airway responsiveness to methacholine differs between KO and WT mice following LPS administration. Notably, our knockout mice showed decreased tissue damping and tissue elastance response to methacholine following LPS exposure compared to WT, indicating an attenuated response to inflammation. ADAM19 facilitates the release of TNF from the cell membrane, promoting an inflammatory response and contributing to the development of inflammatory diseases [17, 18, 37, 58]. We did not identify genotype differences of cytokine changes following LPS (vs. saline). This could reflect the limited number of cytokines examined, which is a limitation of our study. However, GSEA identified the enrichment of downregulated differentially expressed genes in TNF signaling pathways in our *Adam19* KO mice. This is consistent with these previous findings [17, 18, 37, 58] and helps explain the reduced lung functional response to the inflammation in our knockout mice.

In summary, we created a viable whole-body *Adam19* knockout and used this model to examine the role of *Adam19* in lung function, following up on findings from human GWAS implicating this gene. In addition to smaller body size, the lack of functional *Adam19* resulted in reduced respiratory system elastance, minute work of breathing, tissue elastance, forced expiratory flow at 50% FVC, and increased FEV_0.1_ and FVC. Pathway analysis of genes differentially expressed after disruption of *Adam19* implicates pathways crucial in lung inflammation, including TNF signaling pathways. Our data provide evidence to support a causal role for *Adam19* in regulating pulmonary function development. Although our study is limited to a descriptive scope and a definitive understanding of mechanisms underlying our findings requires further investigation, our novel *Adam19* KO murine model could be helpful in future studies to dissect the role of this gene in lung function.

## Supplementary Information

Below is the link to the electronic supplementary material.

Supplementary file1 (DOCX 101 KB)

Supplementary file2 (PDF 4669 KB)

## Data Availability

No datasets were generated or analyzed during the current study.
