# Supplementary material for: Adam19 Deficiency Impacts Pulmonary Function: Human GWAS Follow-up in a Mouse Knockout Model
Source: Lung. 2024 Aug 17;202(5):659–72. doi: 10.1007/s00408-024-00738-7 (PMC11427501; doi:10.1007/s00408-024-00738-7)
Supplement: Supplementary file 1 — Supplementary file1 (DOCX 101 KB) [file 408_2024_738_MOESM1_ESM.docx]

***Adam19* Deficiency Impacts Pulmonary Function: Human GWAS Follow-up in a Mouse Knockout Model**

Huiling Li, John S. House, Cody E. Nichols, Artiom Gruzdev, James M. Ward, Jian-Liang Li, Annah B. Wyss, Ezazul Haque, Matthew L. Edin, Susan A. Elmore, Beth W. Mahler, Laura M. Degraff, Min Shi, Darryl C. Zeldin, and Stephanie J. London

**Online Supplementary Information**

**Supplementary Materials and Methods**

***Adam19* Gene Targeting Scheme and Murine Studies**

The mouse *Adam19* gene has four transcript isoforms, the longest consisting of 23 exons. It encodes the ADAM19 protein (Fig. 1). We originally designed the Adam19 flox allele with loxp sites that would allow for recombinase-mediated cassette exchange in addition to the traditional conditional null characteristics found in a typical flox allele. To generate the Adam19-tdTomato null allele, we utilized a cre-mediated cassette exchange to replace the floxed exons 6 and 7 with the in-frame tdTomato construct. We replaced exons 6 and 7 with an in-frame tdTomato open reading frame -polyA cassette and an antisense hygromycin resistance cassette, resulting in a truncated ADAM19 protein lacking the active catalytic site. The homozygous *Adam19-dTomato* allele is equivalent to the canonical *Adam19*-deficient allele or *Adam19* KO. Our Adam19-deficient allele was generated in 129S ES cells and maintained on the 129S6/SvEvTac background.

To confirm the genetic background of the mice used in this study, we submitted a small set of mouse tail biopsies to Transnetyx (Cordova, TN, USA) for genetic monitoring analysis run on the NeoGen miniMUGA platform (Lansing, MI, USA) [1]. Four familial mice were submitted for whole genome SNP scan: one wildtype (WT), two heterozygous *Adam19-tdTomato* (HET), and one homozygous *Adam19-tdTomato* (KO) for the null allele. Of the 10,819 SNPs on the miniMUGA platform, all four mice had the same SNP call for 10,314 SNPs (95.3%) and consistent with expected results for 129S6/SvEvTac mice. A small cluster of five SNPs around the Adam19 locus had the expected pattern between wildtype, heterozygous, and homozygous mutant, which is due to the original allele being generated in AB2.2 ES cells from 129S5/SvEvBrd mice and subsequently backcrossed to 129S6/SvEvTac mice. Of the differentially called autosomal SNPs, 212 out of 414 were differentially called due to sample quality issues, resulting in no SNP call for a particular position. The remaining 202 differential SNPs (1.9% of all SNPs on the miniMUGA platform) were distributed throughout the genome. Based on these results and mouse colony breeding practices, we are confident that the genetic background of the mice in our study is 129S6/SvEvTac, except for a small region around the *Adam19-tdTomato*/null locus, which is 129S5.

Heterozygous *Adam19-TdTomato* mice were bred to each other to generate *Adam19* KO for experiments; the WT littermates were used as controls. Mouse genotypes were determined by Transnetyx. Mice were fed NIH-31 rodent chow ad libitum and housed with alternating 12-hour light/dark cycles. The mice in this study were all male and aged 9-13 weeks. Two studies found that male mice are more responsive to methacholine challenge and LPS [1, 2]. Therefore, to reduce noise, we used males. We are not aware of evidence that human GWAS results for ADAM19 lung function are sex dependent. However, current directives from NIH encourage the study of both sexes; thus, using males only is a limitation of our study. All animal work described in this study was conducted according to NIH guidelines and approved by the NIEHS Animal Care and Use Committee.

**RNA-Seq and RT-qPCR**

*Adam19* WT and KO mice (n=3 each genotype) were euthanized with an overdose of sodium pentobarbital (200 mg/kg, i.p. injection) followed by puncture of the diaphragm and vena cava exsanguination. The whole heart and lung were immediately removed and frozen with liquid nitrogen. RNA was isolated from the heart and lung using the RNeasy® Mini Kit (Qiagen, Hilden, Germany). RNA-Seq strand-specific libraries were prepared (n=3 per genotype per tissue), and sequencing was conducted on Illumina NextSeq with > 50 million stranded 100 bp pair-end reads obtained per sample. RNA transcript reads were quantified versus GENCODE vM33 comprehensive transcripts (mm39) using Salmon 1.10.0 [2]. The R package "splicejam" (https://github.com/jmw86069/splicejam) was used to count splice junction reads, visualize sequence coverage, and generate sashimi plots [3].

For the RT-qPCR assays, cDNA libraries were prepared using the High-Capacity cDNA Reverse Transcription Kit (Thermo Fisher). *Adam19* exon expression was assessed with TaqMan assay (Thermo Fisher) and *tdTomato* red gene mRNA expression by SYBR green assay using ABI 7900HT (Applied Biosystems). Expression values are represented relative to mouse glyceraldehyde 3-phosphate dehydrogenase (*Gapdh*) expression and normalized to WT (n=4 per genotype per tissue).

**Immunohistochemistry staining of td-TOMATO protein in mouse lung tissue**

*Adam19* WT and HET mice were euthanized with an overdose of sodium pentobarbital (200 mg/kg, i.p. injection) followed by puncture of the diaphragm and vena cava exsanguination. Whole lungs were removed, and lung sections were stained with Anti-tdTomato [16D7] antibody (Catalog No: EST203, Kerafast, Inc., Shirley, MA, USA).

**Differential Gene Expression and Gene Set Enrichment Analysis**

*Adam19-tdTomato* transcripts were excluded during transcript quantification by Salmon. Raw transcript pseudo counts from Salmon were log-transformed and normalized by log-ratio normalization. We used the normalized count data as recommended to conduct the differential gene expression analysis using limma-voom [1,5]. We reported the max group mean (MGM) for the comparison. Statistically significant hits were required to have Benjamini Hochberg adjusted *p*-value < 0.05, fold change > 1.5, and at least one group mean, represented as MGM, at or above 6 log2 counts.

Gene set enrichment analyses (GSEA) [3] of the RNA-seq expression data were performed against the Broad Molecular Signature Database (MSigDB, v2023.2.Mm) to determine potential enriched pathways in *Adam19* RNA-Seq datasets. Genes were ranked based on their differential expression values between *Adam19* KO and WT control, and 1000 permutations were applied to assess the significance level of gene set enrichment. The analysis was performed against the collection of MSigDB hallmark gene sets. Gene sets with FDR *p-value* < 0.25 were defined as significantly enriched.

**Embryo Organogenesis**

For the organ morphogenesis analysis, E18.5 embryos were collected, and serial consecutive sagittal sections (5 µm) were cut through whole embryos. We prepared the heart to have all four heart chambers visible. The tissue slides were stained with Hematoxylin and Eosin (H&E). The morphology of the embryo tissue sections was evaluated by a pathologist, paying thorough attention to the heart for tetralogy of Fallot characteristics (overriding aorta, pulmonic stenosis, ventricular septal defect, right ventricular hypertrophy), lung anatomic structure (trachea, bronchi, bronchioles, alveoli, etc.), adrenal gland, and diaphragm.

**Assessment of Body Weight, Tibia Length, and Body Composition**

Mice (n=248) were weighed by a standard top-loading scale with an accuracy of 0.01g, including all three genotypes (wild-type, knockout, and heterozygote). Mice (n=44) were euthanized with Fatal Plus, and the tibia bones were isolated and measured using a ruler with 0.1 cm accuracy. A subset of 20 mice was assessed for body composition parameters [4] using the Faxitron Dual-energy X-ray Absorptiometry (DXA) imaging system (UltraFocus DXA, Tucson, Arizona, US) following the manufacturer's instructions. Briefly, mice 9-12 weeks of age were anesthetized with isoflurane and placed prone inside the DXA analyzer for X-ray scanning at dual-energy levels [4]. The following parameters were assessed: bone mineral content (BMC), bone mineral density (BMD), bone area, fat weight, fat % (the ratio of fat weight/soft weight in percentage), lean weight, soft weight (fat weight + lean weight), total weight (soft weight + bone mineral content), and sample area indicating the mouse size.

**Pulmonary Function Parameter Measurements in Mice**

Baseline pulmonary function parameters were assessed in anesthetized (urethane 2 g/kg, i.p.), diaphragm paralyzed (0.8 mg/kg pancuronium bromide), tracheotomized (using an 18G x ½" cannula) mice connected to a computer-controlled piston ventilator FX2 with negative pressure-driven forced expiratory extension (SCIREQ Scientific Respiratory Equipment Inc., Montréal, Canada) as previously described [5, 6]. The flexiVent system uses the forced oscillation technique (FOT) to apply airwaves to measure pressure, flow, and volume parameters in the lung in response to the input signals [7, 8]. According to the manufacturer's instructions, when determining lung function using flexiVent, we input the mouse's weight to adjust the volume-driven perturbations. Additionally, the manufacturer stated that the analysis of the raw signals during forced oscillation is based on phase shifts rather than differences in amplitude. Furthermore, we adjusted all statistical analyses of lung function parameters for weight to ensure that any observed differences in lung function were not due to the animals' sizes but instead to Adam19 genetic deficiencies. We also used an extension of the flexiVent system negative pressure-driven forced expiratory (NPFE) concurrently to apply a negative pressure (-55 cmH2O) to deflate the lung rapidly. Then, we recorded the response [6]. The resulting measurements mimic human spirometry, including the forced expired volume over 0.1second (FEV0.1), forced vital capacity (FVC), FEV0.1/FVC, forced expiratory flow at 50% of FVC (FEF50), Forced Expired Volume at Peak Expiratory Flow (FEV_PEF), and peak expiratory flow (PEF). In addition to baseline measurements, airway responsiveness was induced with methacholine (administered via nebulizer at each dose of 0, 3, 10, 30, and 100 mg/ml in PBS), and pulmonary function parameters were again assessed at each methacholine dose.

For LPS experiments, mice were given LPS (Sigma, L-2630) or saline (pharmaceutical grade) via oropharyngeal aspiration. Four hours post the exposure treatment, the baseline lung function and airway responses to methacholine doses (0, 1, 3, 10, and 30 mg/ml in PBS) were measured using the same flexiVent procedure.

**Lung Histology Examination**

To better understand the lung tissue structure behind the lung function phenotype, we evaluated if the pulmonary collagen content and extracellular matrix were altered in *Adam19* knockout mice. The collagen content was investigated with Masson’s trichrome (MT), and extracellular matrix accumulation was studied with periodic acid-Schiff (PAS) special stains. Lungs from WT and KO naïve mice were lavaged before fixation, processed, and stained with hematoxylin and eosin (H&E), MT, and PAS stains. Sections stained with H&E were not evaluated but used as reference only. The collagen content was assessed using MT- and PAS-stained lung sections.

**Immune Cell Profile and Cytokine Analysis in BALF in Naïve and LPS Exposed Mice**

Mice were euthanized with Fatal Plus, and airways were rinsed with 1 ml of Hanks Buffered Salt Solution (HBSS; H6648; Sigma-Aldrich, St. Louis, MO) twice. Bronchial alveolar lavage fluid (BALF) from each mouse was pooled, and red blood cells were lysed with ammonium-chloride-potassium buffer [5]. Cell counts were quantified with an automated cell counter TC20™ (Bio-Rad, Hercules, CA, USA), and cytospins were prepared for cell differential analysis.

For LPS or saline exposure, 50 μl of either 50 μg bacterial LPS (Escherichia coli O111: B4; L2630; Sigma-Aldrich, St. Louis, MO, USA) or endotoxin-free saline (S8776 [certificate 7647-14-5 (<0.005 U/ml endotoxin)]; Sigma-Aldrich) was administered via oropharyngeal aspiration to Adam19 KO and WT mice post-anesthetization with isoflurane/oxygen. Mice were monitored hourly for body temperature and signs of distress, and BALF collection was performed at 4 hours post-administration and analyzed as described above.

Aliquots of the above BALF were kept in a -80C freezer. One aliquot of each BALF sample was used for cytokine detection. A custom Bio-Plex Pro Mouse Cytokine 8-plex Assay was obtained from Bio-Rad. The manufacturer’s instructions were followed to determine the concentrations of the following cytokines: IL-1b, IL-2, IL-6, KC, MCP-1, MIP-1a, MIP-1b, and TNF-a.

**Statistical Analyses**

We analyzed body weight, body composition parameters, and tibia length using linear regression adjusting for weeks of age because mice may still be growing at ages of measurement (9-13 weeks). We used a general linear model to analyze the genotype difference for each lung function parameter at baseline for the untreated mice and mice treated with LPS. For airway responsiveness to methacholine doses, we fit the data from the methacholine concentrations using a linear mixed-effect model with a random intercept for each animal. Response variables were normalized using the corresponding values at PBS, and the natural logarithm of the dose level was used as a predictor in the model, along with genotype or genotype and treatment. We also included all the two-way interactions between genotype, treatment, and log dose and the three-way interactions. This three-way interaction term allows us to assess the difference in slope changes due to LPS between the wild-type and the knockout mice. All lung function parameters were adjusted for body weight. The experimental date was adjusted as a categorical covariate to account for potential confounding due to batch effects in both general linear regression and the linear mixed-effect model. P values for lung function were adjusted by the Sidak method [9]. Because of weight differences by genotype, all lung function parameters were analyzed with bodyweight adjustment. One animal with extreme outlying values of total macrophages identified with Tukey’s fences method was excluded from differential cell count analyses. Linear regression was used to analyze treatment (LPS vs. saline) effects across genotypes with log-transformed total cell counts or log-transformed macrophage counts as the response variable, experiment date, treatment, genotype, and the interaction between treatment and genotype as the predictors. Due to the presence of zero values, neutrophil counts were not log-transformed. Instead, a robust linear model was implemented in the “estimatr” R package to analyze the genotype difference responding to the treatment (LPS vs. saline) [10]. For lymphocytes, due to the lower percentage of above zero counts, we dichotomized lymphocyte counts to 0 and > 0 and used logistic regression in testing association with lymphocytes > 0. Due to the absence of eosinophils except in one mouse, we did not analyze eosinophil data. For the cytokine data, linear regression with a robust sandwich estimator was used in the analysis. Original measured values were used as the response variable. Machine extrapolated values were used directly. Values were treated as missing if under the lower detection limit of the assay. For cytokine levels in the untreated mice, genotype differences were analyzed with the following predictors: experimental date as a categorical variable, age, and genotype. For saline-treated mice, the genotype difference for each cytokine was assessed using additional predictors, such as treatment and treatment-genotype interaction. All the analyses and figures were done using R version 4.2.2.

**Supplementary References**

1. Sigmon, J.S., M.W. Blanchard, R.S. Baric, et al., *Content and Performance of the MiniMUGA Genotyping Array: A New Tool To Improve Rigor and Reproducibility in Mouse Research.* Genetics, 2020. **216**(4): p. 905-930.

2. Patro, R., G. Duggal, M.I. Love, et al., *Salmon provides fast and bias-aware quantification of transcript expression.* Nat Methods, 2017. **14**(4): p. 417-419.

3. Subramanian, A., P. Tamayo, V.K. Mootha, et al., *Gene set enrichment analysis: A knowledge-based approach for interpreting genome-wide expression profiles.* Proceedings of the National Academy of Sciences, 2005. **102**(43): p. 15545-15550.

4. Nagy, T.R. and A.-L. Clair, *Precision and Accuracy of Dual-Energy X-ray Absorptiometry for Determining in Vivo Body Composition of Mice.* Obesity Research, 2000. **8**(5): p. 392-398.

5. House, J.S., H. Li, L.M. DeGraff, et al., *Genetic variation in HTR4 and lung function: GWAS follow-up in mouse.* Faseb j, 2015. **29**(1): p. 323-35.

6. Shalaby, K.H., L.G. Gold, T.F. Schuessler, et al., *Combined forced oscillation and forced expiration measurements in mice for the assessment of airway hyperresponsiveness.* Respir Res, 2010. **11**: p. 82.

7. Bates, J.H.T., *CORP: Measurement of lung function in small animals.* Journal of Applied Physiology, 2017. **123**(5): p. 1039-1046.

8. Irvin, C.G. and J.H.T. Bates, *Measuring the lung function in the mouse: the challenge of size.* Respiratory Research, 2003. **4**(1): p. 1.

9. Sidak, Z., *Rectangular Confidence Regions for the Means of Multivariate Normal Distributions.* Journal of the American Statistical Association, 1967. **62**(318): p. 626-633.

10. Lin, W., *Agnostic Notes on Regression Adjustments to Experimental Data: Reexamining Freedman's Critique.* Annals of Applied Statistics, 2013. **7**(1): p. 295-318.

**Supplementary Figure Legends**

**Fig. S1 *Adam19* KO RNA-seq transcript alignment on the UCSC genome browser.** A: The orange color represents the heart, and the purple color represents the lung. *Adam19* exon 1-5 were expressed in both the hearts and lungs. The tdTomato construct, shown as an orange box, was inserted into the targeted region to replace exons 6 and 7, disrupting the Adam19 gene. No transcripts were expressed from exon 8 through the end of the *Adam19* gene. These sequence tracks can be viewed by the general public via the NIEHS-hosted track hub with UCSC Genome Browser by entering the URL <https://genome.ucsc.edu/cgi-bin/hgTracks?genome=mm39_adam19tdt&hubUrl=https://orio.niehs.nih.gov/ucscview/Adam19/hub.txt>. B: The region containing tdTomato is enlarged for more detail. *Adam19_tdTomato_tx*: *Adam19 tdTomato* transcript; HygroR=Hygromycin Resistance. CAG: CMV enhancer, chicken beta-Actin promoter, and rabbit beta-Globin splice acceptor site.

**Fig. S2 RT-qPCR of *Adam19* exon transcripts by TaqMan® and tdTomato mRNA by SYBR Green methods.** The number in the x-axis indicates the exon boundary spanned by each TaqMan® primer/probe. dTom= tdTomato red gene; *Gapdh*=glyceraldehyde 3-phosphate dehydrogenase; nd=not detected; n=4 mice per genotype per tissue.

**Fig. S3 Anti-tdTOMATO immunohistochemistry (IHC) was conducted on lung tissue of *Adam19* WT (A and B) and *Adam19-tdTomato* heterozygous mice (C and D).** *Adam19* WT mice with primary antibody (A) and without (B): Arrows indicate non-specific staining. IHC was conducted on heterozygous *Adam19-tdTomato* mice with primary antibody (C) and without (D): Arrows in (C) indicate positive staining in the alveolar type-II epithelium (based on the morphology and location), and no staining was present in the negative control section. The microscope magnifications for each panel were 40X. HET: heterozygous *Adam19-tdTomato*

**Fig. S4 Heat map of differential gene expression for genes significantly differentially expressed between *Adam19* KO and WT in the lung.** Significant differential expression was based on the following criteria: adjusted p-value ≤ 0.05, absolute fold change ≥ 1.5, and means maximum group mean (the highest normalized group mean abundance for each gene) ≥ 6. Each column represents a sample; each row represents a gene. Centered expression means the difference in the expression fold change level between each KO and WT mean. n=3 per genotype.

**Fig. S5 Hematoxylin and eosin staining of mouse E18.5 embryo organ tissues.** The cellular and tissue structures of hearts, lungs, pulmonary valves, adrenal glands, and diaphragms were evaluated by a pathologist, and no abnormalities were found. The microscope magnifications for each tissue structure were 4X for hearts, 20X for lungs, pulmonary valves, and adrenal glands, and 40X for diaphragms.

**Fig. S6 *Adam19* KO mice had stopped gaining weight by nine weeks, whereas WT continued to grow—bodyweight vs. age.** WT mice continued to gain weight from 9 to 12 weeks (β = 0.7928 grams/week, 95% confidence interval = 0.2543-3.117, *p* = 0.00254), whereas the KO mice had stopped gaining weight by nine weeks (*p* = 0.937). WT: n=114, KO: n=104.

**Fig. S7 *Adam19* gene disruption does not alter airway responsiveness to methacholine.** The maximum response to MCH at each dose was expressed as a percentage of the maximum response at PBS. Means and standard errors of means are indicated as bar lines. R_rs_=resistance of the respiratory system; E_rs_=elastance of the respiratory system; mWOB=minute work of breathing; R_N_=Newtonian resistance; G=tissue damping; H=tissue elastance; BL=Baseline; PBS=phosphate buffered saline. %PBS=maximum response to methacholine at each dose as a percentage of the maximum response at PBS. n=22 for WT; n=15 for KO.

**Fig. S8 *Adam19* deficiency does not alter (A) baseline mechanic and (B) spirometry parameters determined by flexiVent following the exposure to LPS.** R_rs_=resistance of the respiratory system; E_rs_=elastance of the respiratory system; mWOB=minute work of breathing; J=joule (one joule is the work required to move 1 liter of gas through a 10-cmH_2_O pressure gradient). R_N_= Newtonian resistance; G=tissue damping; H=tissue elastance; FEV_0.1_=forced expiratory volume in 0.1 s; FVC=forced vital capacity; FEV_0.1_/FVC=the ratio of FEV_0.1_ over FVC in %; FEF50=Forced expiratory flow at 50% FVC; FEV_PEF=Forced expiratory volume at peak expiratory flow; PEF=Peak expiratory flow. n=13 for WT-Saline; n=20 for WT-LPS; n=14 for KO-Saline; n=21 for KO-LPS.

**Fig. S9 Collagen content and connective tissue in wildtype (A, C) and *Adam19* KO (B, D) mice.** Masson's Trichrome (A, B) stains collagen fibers in blue and cells in red; Periodic Acid-Schiff stains extracellular matrix and basal laminae in reddish-purple. There is no evidence of high collagen fiber density or deposition in any of the interstitium (arrowheads), surrounding airways (arrows), or alveoli (asterisks) in *Adam19* KO mice when compared to wildtype mice. The microscope magnifications for each panel were 40X. MT=Masson's Trichrome; PAS=Periodic Acid-Schiff.

**Fig. S10 Cytokine levels in bronchoalveolar lavage fluid (BALF) in mice following LPS exposure.** The increased degrees of cytokines (IL-6, KC, MCP-1, MIP-1a, MIP-1b, and TNF) following LPS (vs. saline) were not different in the *Adam19* KO and WT mice. SAL=Saline. WT: n=10 (SAL), 10 (LPS); KO: n=7 (SAL), 8 (LPS). Y axes are presented in log scale. All *p* values for differences by genotypes of cytokine changes following LPS (vs. saline) were greater than 0.05.

**Table S1 Biological pathways enriched significantly (FDR < 0.25) in *Adam19* KO lung compared to WT. Broad Molecular Signature Database (MSigDB v2023.2.Mm) hallmark gene sets collection was used.**

| **Hallmark Name** | **Number of Genes** | **NES^a^** | ***p*-value** | **FDR** |
| --- | --- | --- | --- | --- |
| MYC_TARGETS_V1 | 192 | -3.412 | < 0.001 | < 0.001 |
| OXIDATIVE_PHOSPHORYLATION | 193 | -3.060 | < 0.001 | < 0.001 |
| E2F_TARGETS | 162 | -2.583 | < 0.001 | < 0.001 |
| UNFOLDED_PROTEIN_RESPONSE | 109 | -2.171 | 0.004 | 0.012 |
| PROTEIN_SECRETION | 91 | -1.857 | 0.008 | 0.069 |
| TNFA_SIGNALING_VIA_NFKB | 165 | -1.808 | 0.020 | 0.077 |
| G2M_CHECKPOINT | 161 | -1.708 | 0.022 | 0.107 |
| DNA_REPAIR | 139 | -1.644 | 0.030 | 0.126 |
| MITOTIC_SPINDLE | 181 | 1.933 | 0.010 | 0.170 |

Definition of abbreviations: NES = normalized enrichment score; FDR = false discovery rate.

^a^NES>0 indicates positive enrichment, meaning enrichment by up-regulated genes. NES<0 indicates negative enrichment, meaning enrichment by down-regulated genes. The table was sorted by NES ascendingly.

**Table S2 Body composition parameter values by genotype as shown in Fig. 2C-L**

|  | WT (n=10) | |  | KO (n=9) | |  | |
| --- | --- | --- | --- | --- | --- | --- | --- |
| Parameters | Mean | SD |  | Mean | SD |  | ***p*-value** |
| Body Weight (g) | 24.4 | 1.7 |  | 20.6 | 1.8 |  | 2.03e-04 |
| Sample Area (cm^2^) | 24.0 | 1.3 |  | 20.9 | 1.6 |  | 2.18e-04 |
| Bone Area (cm^2^) | 10.3 | 1.4 |  | 9.0 | 1.2 |  | 0.053 |
| Total Weight (g) | 26.3 | 1.9 |  | 22.4 | 1.9 |  | 0.0004 |
| Soft Weight (g) | 25.5 | 1.9 |  | 21.7 | 1.8 |  | 0.0004 |
| Lean Weight (g) | 18.3 | 2.5 |  | 16.4 | 0.9 |  | 0.056 |
| Fat Weight (g) | 7.2 | 1.3 |  | 5.3 | 1.4 |  | 0.011 |
| Fat Percent (%) | 28.3 | 6.2 |  | 24.3 | 4.5 |  | 0.135 |
| BMC (g) | 0.8 | 0.1 |  | 0.7 | 0.1 |  | 0.023 |
| BMD (mg/cm^2^) | 75.6 | 3.9 |  | 72.3 | 3.1 |  | 0.074 |

SD: standard deviation; BMC: Bone Mineral Content; BMD: Bone Mineral Density = BMC/(Bone Area); Total Weight = Soft Weight + BMC; Soft Weight = Lean Weight + Fat Weight; Fat Percent = (Fat Weight)/(Soft Weight) in percentage. The *p*-values were based on linear regression adjusting for age.

**Table S3 Mean and standard deviation of baseline lung function values by genotype as shown in Fig. 3**

|  | **WT (n=22)** | |  | **KO (n=15)** | | |  |  |
| --- | --- | --- | --- | --- | --- | --- | --- | --- |
| **Parameters** | **Mean** | **SD** |  | **Mean** | **SD** | |  | ***p*-value** |
| **Mechanics** | | | | | | | | |
| R_rs_ (cmH_2_O.s/mL) | 0.79 | 0.13 |  | 0.71 | | 0.08 |  | 0.0542 |
| E_rs_ (cmH_2_O/mL) | 36.98 | 6.33 |  | 31.64 | | 3.37 |  | 0.0016 |
| mWOB (J/L.min) | 125.36 | 9.94 |  | 112.83 | | 5.77 |  | 0.0064 |
| R_N_ (cmH_2_O.s/mL) | 0.20 | 0.06 |  | 0.22 | | 0.06 |  | 0.8867 |
| G (cmH_2_O/mL) | 6.44 | 1.09 |  | 5.73 | | 0.73 |  | 0.0169 |
| H (cmH_2_O/mL) | 33.13 | 5.27 |  | 27.9 | | 3.52 |  | 0.0007 |
| **Spirometry** | | | | | | | | |
| FEV_0.1_ (mL) | 0.81 | 0.10 |  | 0.84 | | 0.06 |  | 0.0218 |
| FVC (mL) | 0.90 | 0.12 |  | 0.93 | | 0.09 |  | 0.0104 |
| FEV_0.1_/FVC (%) | 90.18 | 2.77 |  | 90.23 | | 3.61 |  | 0.4564 |
| FEF50 (mL/s) | 20.61 | 4.63 |  | 15.97 | | 3.20 |  | 0.0172 |
| PEF (mL/s) | 30.10 | 3.88 |  | 30.42 | | 1.85 |  | 0.653 |
| FEV_PEF (mL) | 0.24 | 0.08 |  | 0.22 | | 0.04 |  | 0.7124 |
| **Age and Weight** | | | | | | | | |
| Age (wk) | 10.2 | 0.7 |  | 10.5 | | 0.7 |  | 0.205 |
| Body Weight (g) | 23.3 | 2.0 |  | 21.3 | | 2.5 |  | 0.0118 |

SD-Standard Deviation; R_rs_=resistance of the respiratory system; E_rs_=elastance of the respiratory system; mWOB=minute work of breathing (the work required to breathe in on a minute basis); J=joule (one joule is the work required move 1 liter of gas through a 10-cmH2O pressure gradient); R_N_=Newtonian resistance; G=tissue damping; H=tissue elastance; FEV_0.1_=forced expiratory volume in 0.1 s; FVC=forced vital capacity; FEV_0.1_/FVC = the ratio of FEV_0.1_ over FVC in %; FEF50=Forced expiratory flow at 50% FVC; PEF = Peak expiratory flow; FEV_PEF=Forced expiratory volume at peak expiratory flow.
