# Supplementary material for: Adam19 Deficiency Impacts Pulmonary Function: Human GWAS Follow-up in a Mouse Knockout Model
Source: Lung. 2024 Aug 17;202(5):659–72. doi: 10.1007/s00408-024-00738-7 (PMC11427501; doi:10.1007/s00408-024-00738-7)
Supplement: Supplementary file 2 — Supplementary file2 (PDF 4669 KB) [file 408_2024_738_MOESM2_ESM.pdf]

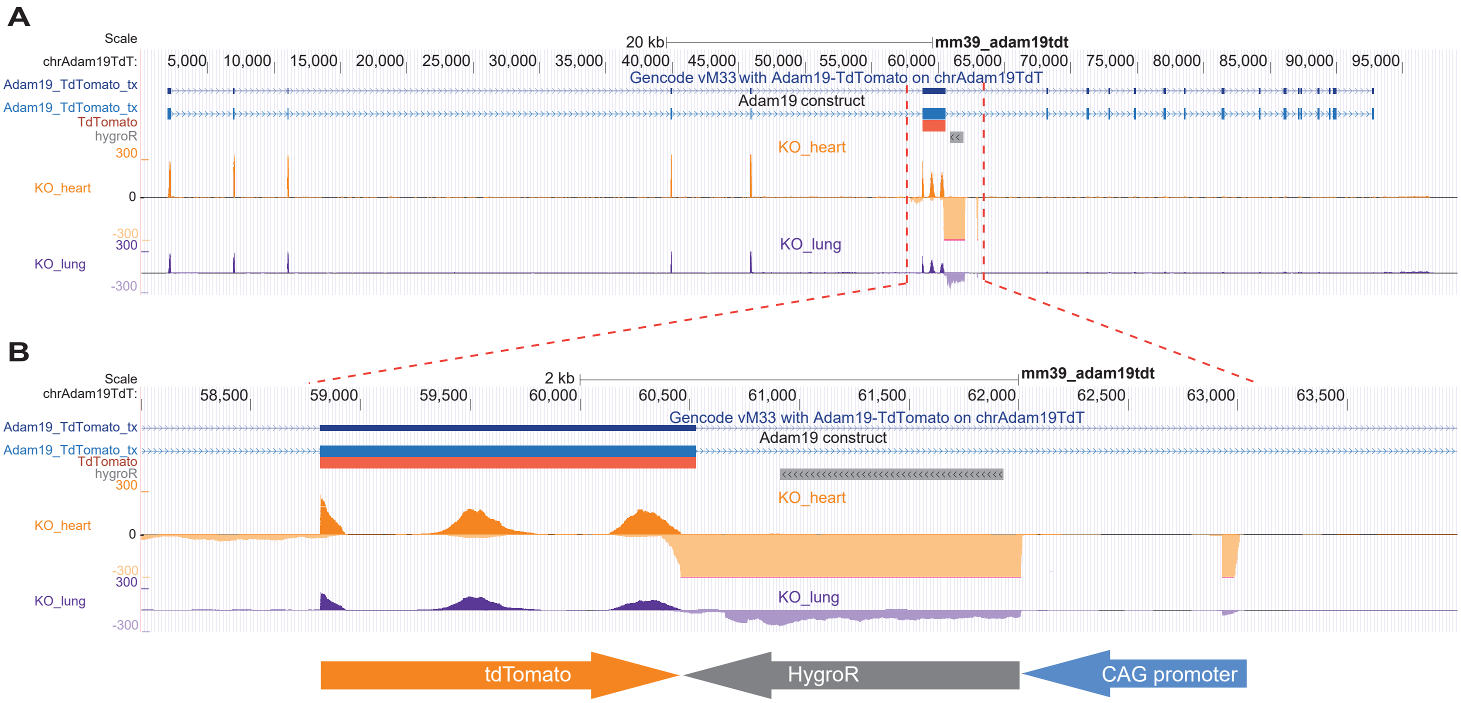


**Fig. S1 *Adam19* KO RNA-seq transcript alignment on the UCSC genome browser.** A: The orange color represents the heart, and the purple color represents the lung. *Adam19* exon 1-5 were expressed in both the hearts and lungs. The tdTomato construct, shown as an orange box, was inserted into the targeted region to replace exons 6 and 7, disrupting the *Adam19* gene. No transcripts were expressed from exon 8 through the end of the *Adam19* gene. These sequence tracks can be viewed by the general public via the NIEHS-hosted track hub with UCSC Genome Browser by entering the URL https://genome.ucsc.edu/cgi-bin/hgTracks?genome=mm39_adam19tdt&hubUrl=https://orio.niehs.nih.gov/ucscview/Adam19/hub.txt. B: The region containing tdTomato is enlarged for more detail. *Adam19_tdTomato_tx*: *Adam19 tdTomato* transcript; HygroR=Hygromycin Resistance. CAG: CMV enhancer, chicken beta-Actin promoter, and rabbit beta-Globin splice acceptor site.

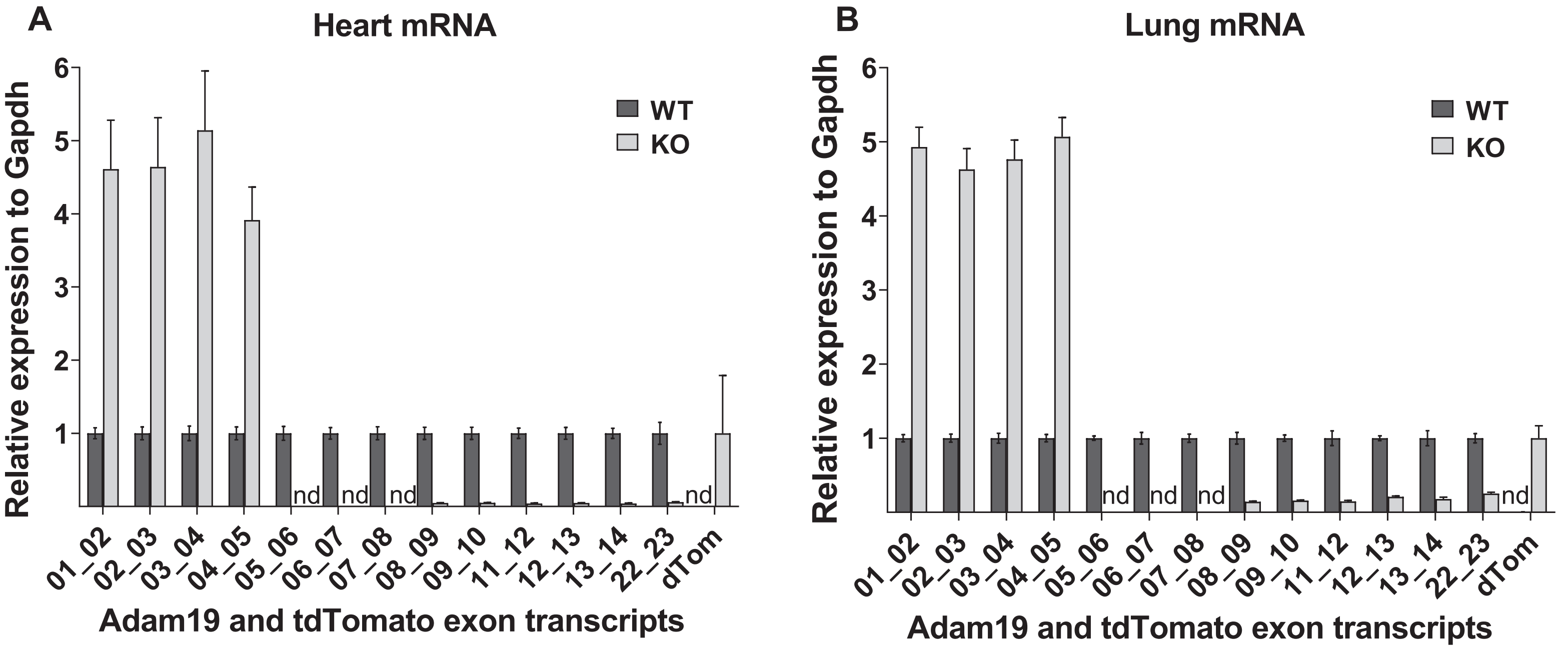


**Fig. S2 RT-qPCR of *Adam19* exon transcripts by TaqMan® and tdTomato mRNA by SYBR Green methods.** The number in the x-axis indicates the exon boundary spanned by each TaqMan® primer/probe. dTom= tdTomato red gene; Gapdh=glyceraldehyde 3-phosphate dehydrogenase; nd=not detected; n=4 mice per genotype per tissue.

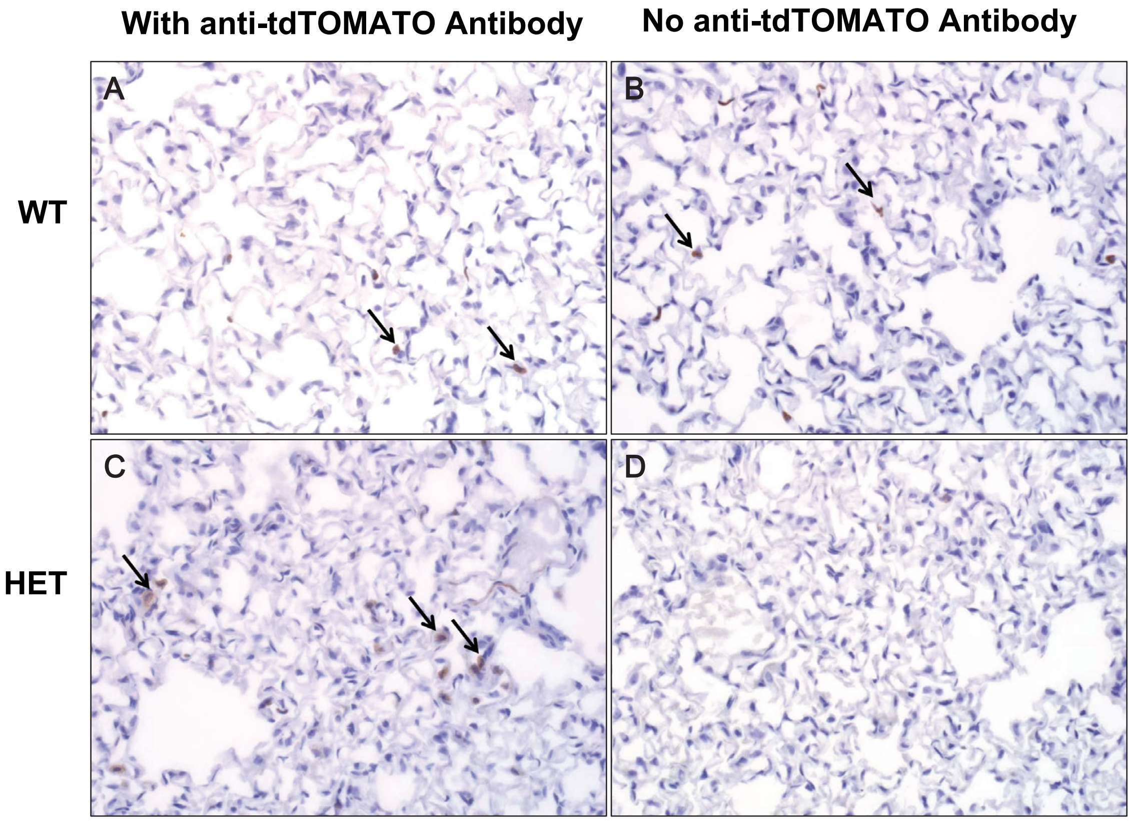


**Fig. S3 Anti-tdTOMATO immunohistochemistry (IHC) was conducted on lung tissue of *Adam19* WT (A and B) and *Adam19-tdTomato* heterozygous mice (C and D).** *Adam19* WT mice with primary antibody (A) and without (B): Arrows indicate non-specific staining. IHC was conducted on heterozygous *Adam19-tdTomato* mice with primary antibody (C) and without (D): Arrows in (C) indicate positive staining in the alveolar type-II epithelium (based on the morphology and location), and no staining was present in the negative control section. The microscope magnifications for each panel were 40X. HET: heterozygous *Adam19-tdTomato*

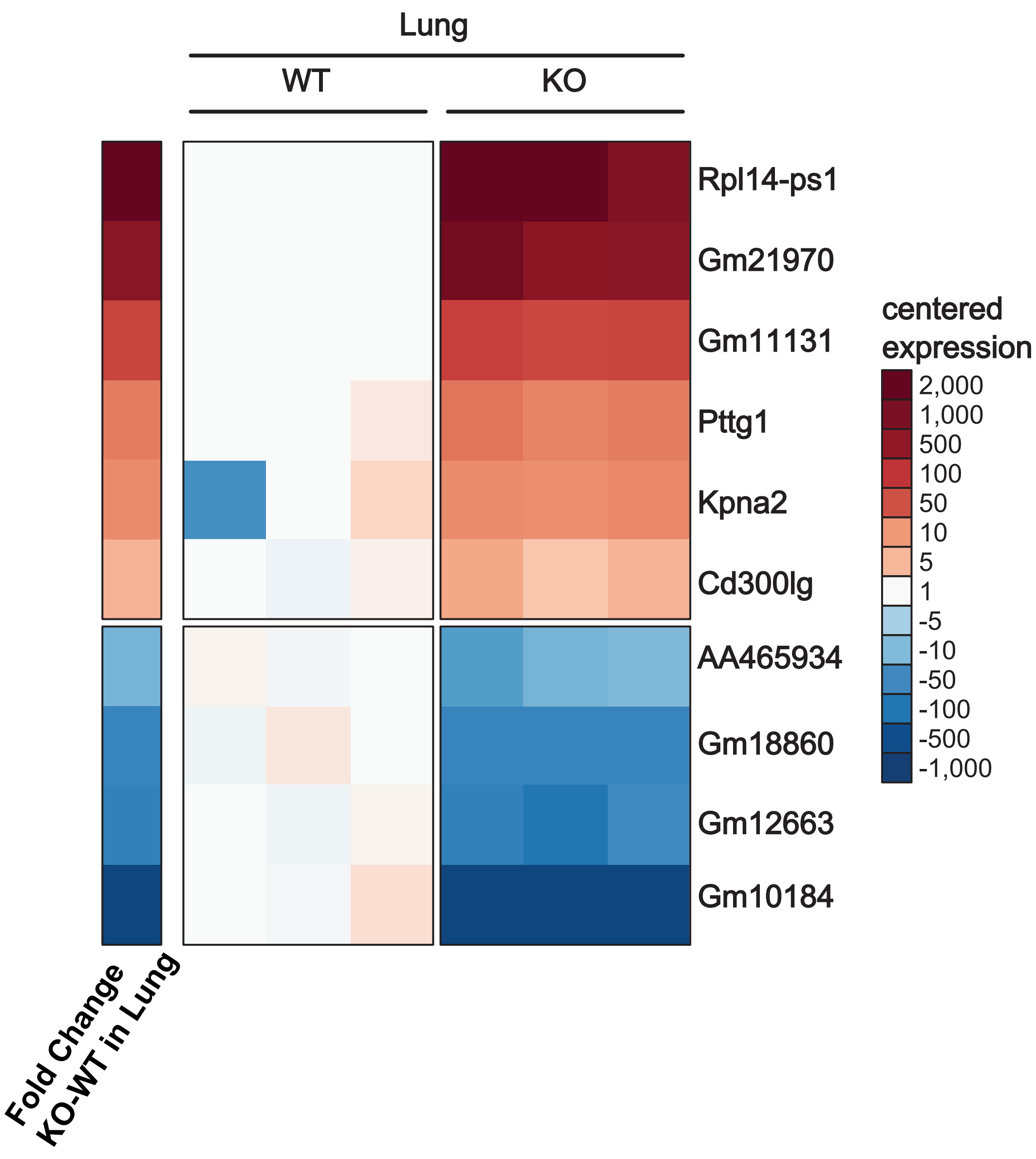


**Fig. S4 Heat map of differential gene expression for genes significantly differentially expressed between *Adam19* KO and WT in the lung.** Significant differential expression was based on the following criteria: adjusted p-value ≤ 0.05, absolute fold change ≥ 1.5, and means maximum group mean (the highest normalized group mean abundance for each gene) ≥ 6. Each column represents a sample; each row represents a gene. Centered expression means the difference in the expression fold change level between each KO and WT mean. n=3 per genotype.

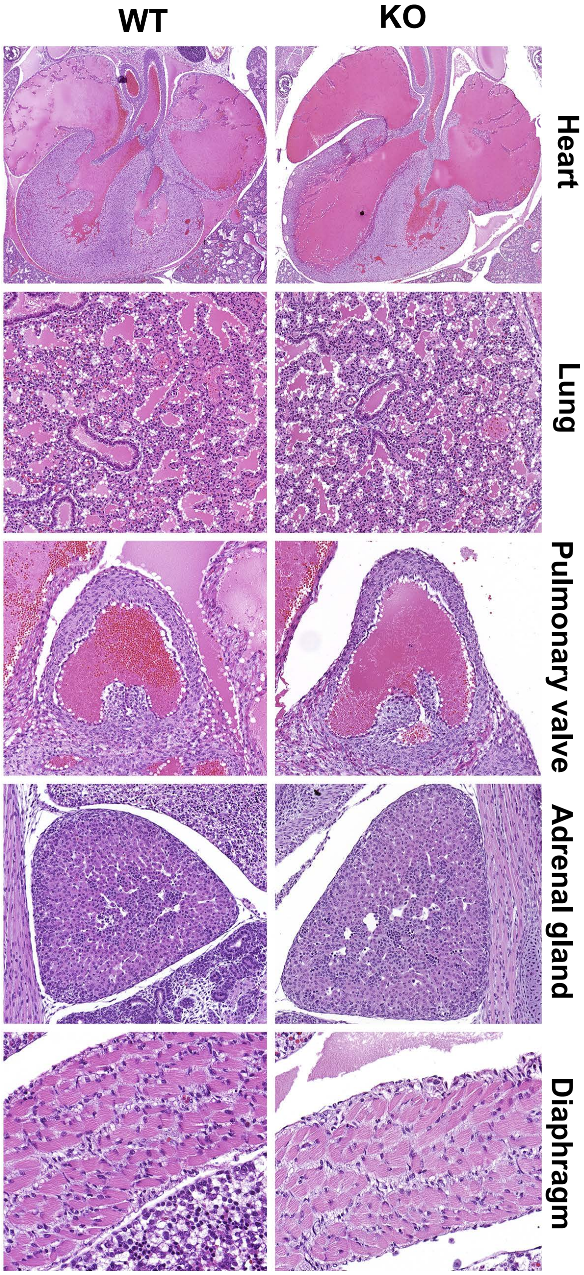


**Fig. S5 Hematoxylin and eosin staining of mouse E18.5 embryo organ tissues.** The cellular and tissue structures of hearts, lungs, pulmonary valves, adrenal glands, and diaphragms were evaluated by a pathologist, and no abnormalities were found. The microscope magnifications for each tissue structure were 4X for hearts, 20X for lungs, pulmonary valves, and adrenal glands, and 40X for diaphragms.

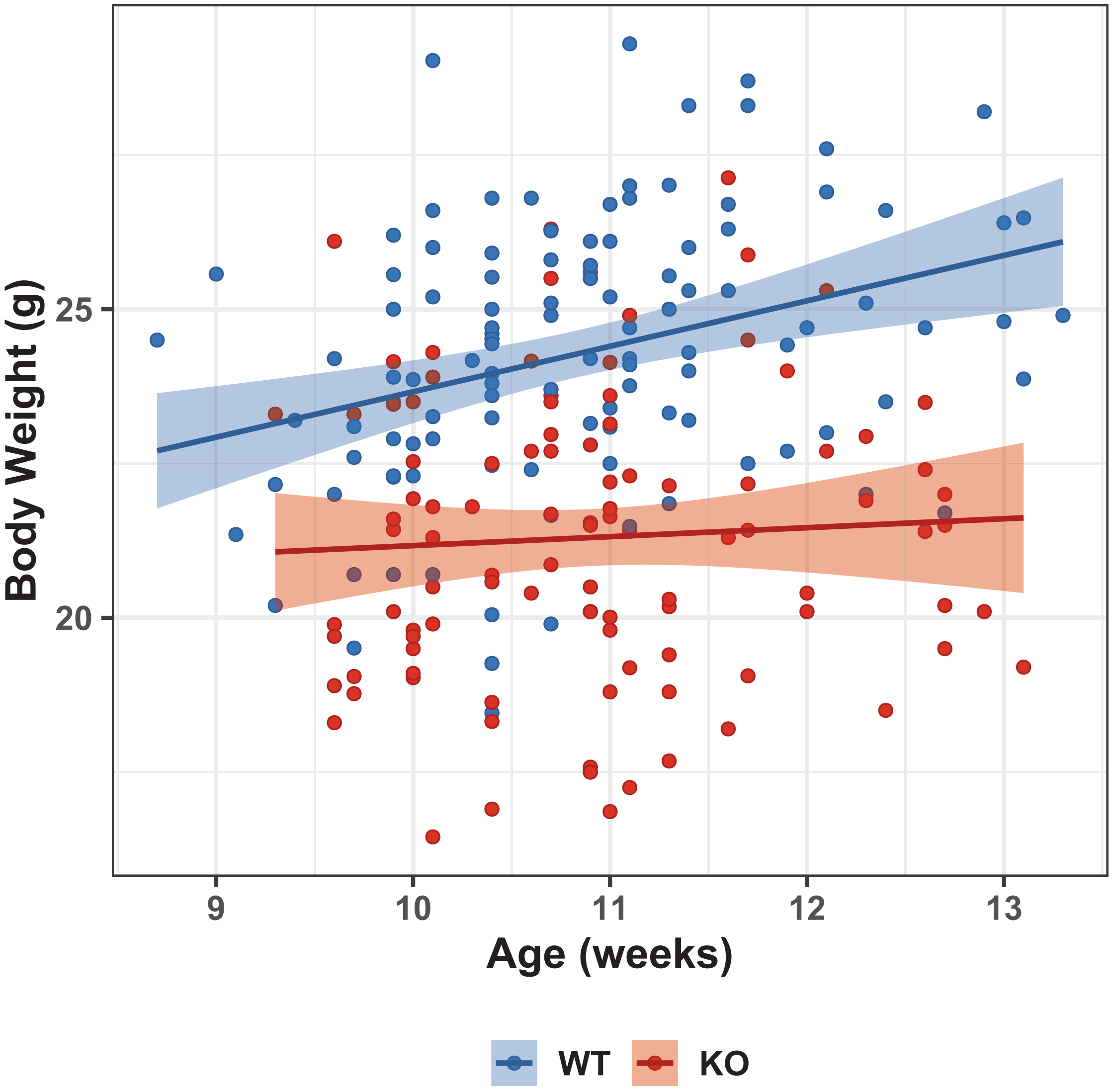


**Fig. S6 *Adam19* KO mice had stopped gaining weight by nine weeks, whereas WT continued to grow—bodyweight vs. age.** WT mice continued to gain weight from 9 to 12 weeks (β = 0.7928 grams/week, 95% confidence interval = 0.2543-3.117, $p$ = 0.00254), whereas the KO mice had stopped gaining weight by nine weeks ($p$ = 0.937). WT: n=114, KO: n=104.

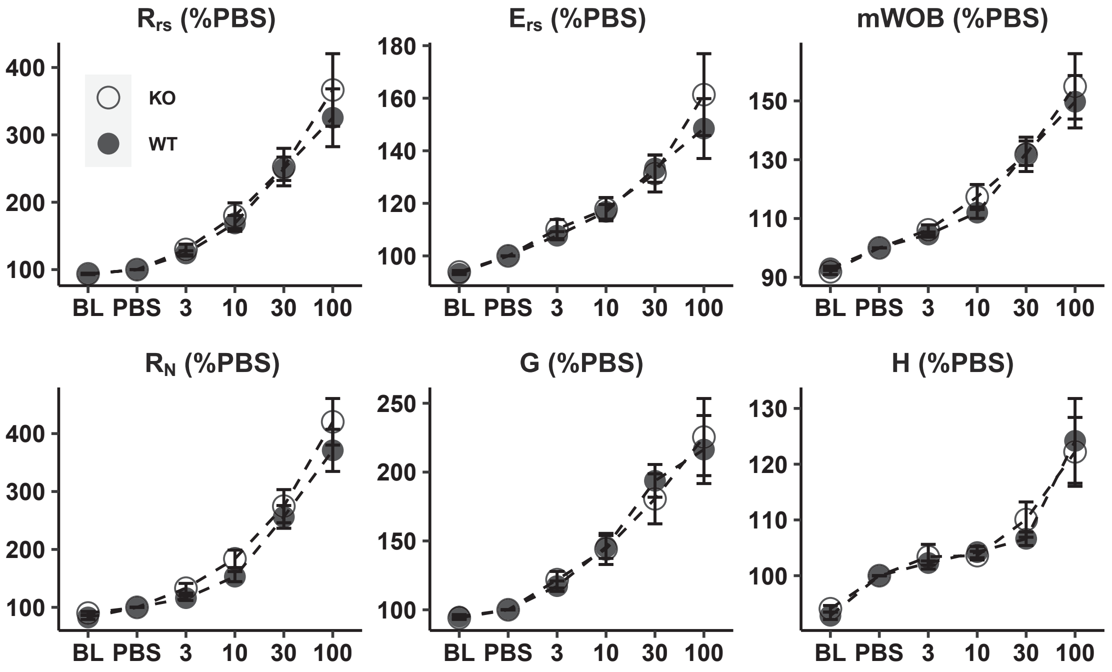


**Fig. S7 *Adam19* gene disruption does not alter airway responsiveness to methacholine.** The maximum response to MCH at each dose was expressed as a percentage of the maximum response at PBS. Means and standard errors of means are indicated as bar lines. $R_{rs}$=resistance of the respiratory system; $E_{rs}$=elastance of the respiratory system; mWOB=minute work of breathing; $R_N$=Newtonian resistance; G=tissue damping; H=tissue elastance; BL=Baseline; PBS=phosphate buffered saline. % PBS=maximum response to methacholine at each dose as a percentage of the maximum response at PBS. n=22 for WT; n=15 for KO.

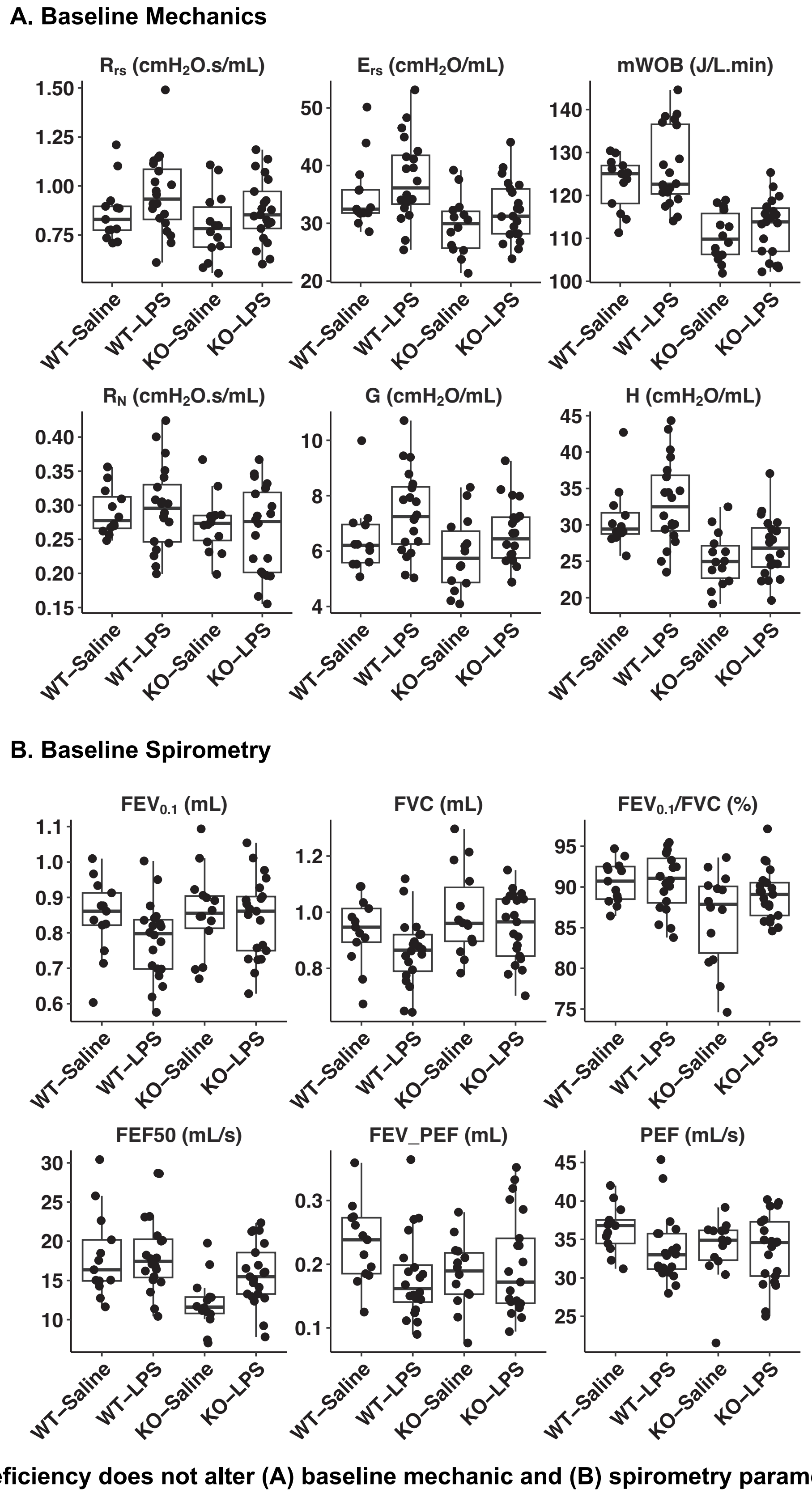


**Fig. S8 *Adam19* deficiency does not alter (A) baseline mechanic and (B) spirometry parameters determined by flexiVent following the exposure to LPS.** $R_{rs}$=resistance of the respiratory system; $E_{rs}$=elastance of the respiratory system; mWOB=minute work of breathing; J=joule (one joule is the work required to move 1 liter of gas through a 10-cm$H_2O$ pressure gradient). $R_N$= Newtonian resistance; G=tissue damping; H=tissue elastance; $FEV_{0.1}$=forced expiratory volume in 0.1 s; FVC=forced vital capacity; $FEV_{0.1}$/FVC=the ratio of $FEV_{0.1}$ over FVC in %; FEF50=Forced expiratory flow at 50% FVC; FEV_PEF=Forced expiratory volume at peak expiratory flow; PEF=Peak expiratory flow. n=13 for WT-Saline; n=20 for WT-LPS; n=14 for KO-Saline; n=21 for KO-LPS.

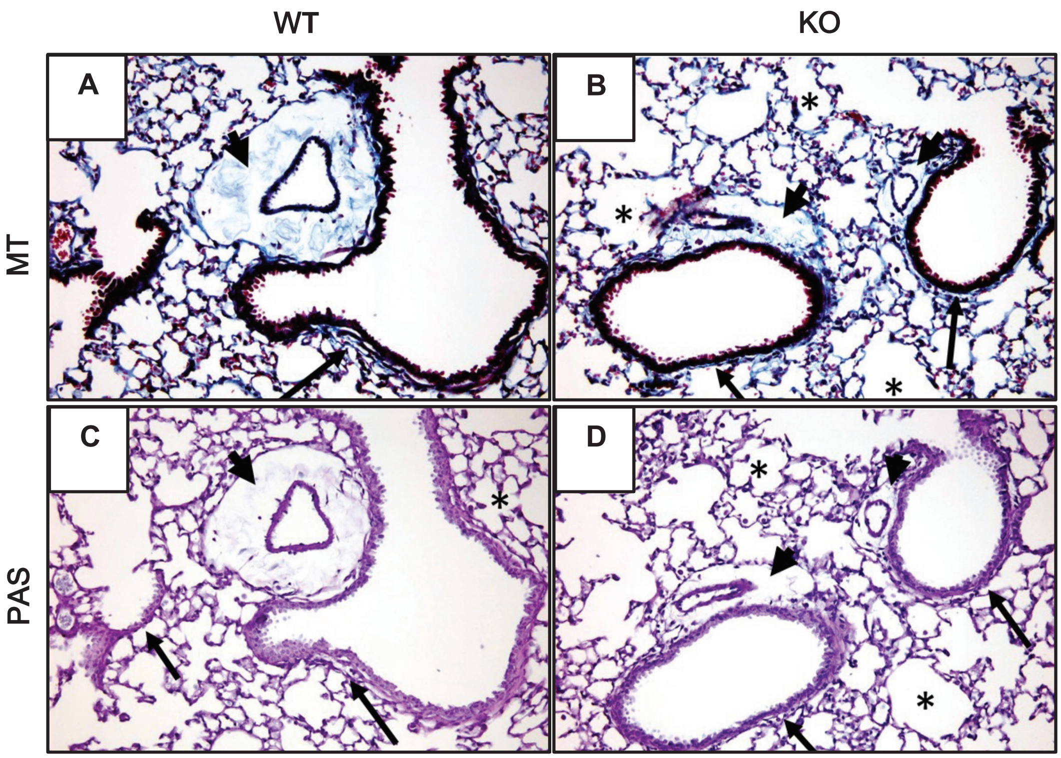


**Fig. S9 Collagen content and connective tissue in wildtype (A, C) and *Adam19* KO (B, D) mice.** Masson's Trichrome (A, B) stains collagen fibers in blue and cells in red; Periodic Acid-Schiff stains extracellular matrix and basal laminae in reddish-purple. There is no evidence of high collagen fiber density or deposition in any of the interstitium (arrowheads), surrounding airways (arrows), or alveoli (asterisks) in *Adam19* KO mice when compared to wildtype mice. The microscope magnifications for each panel were 40X. MT=Masson's Trichrome; PAS=Periodic Acid-Schiff

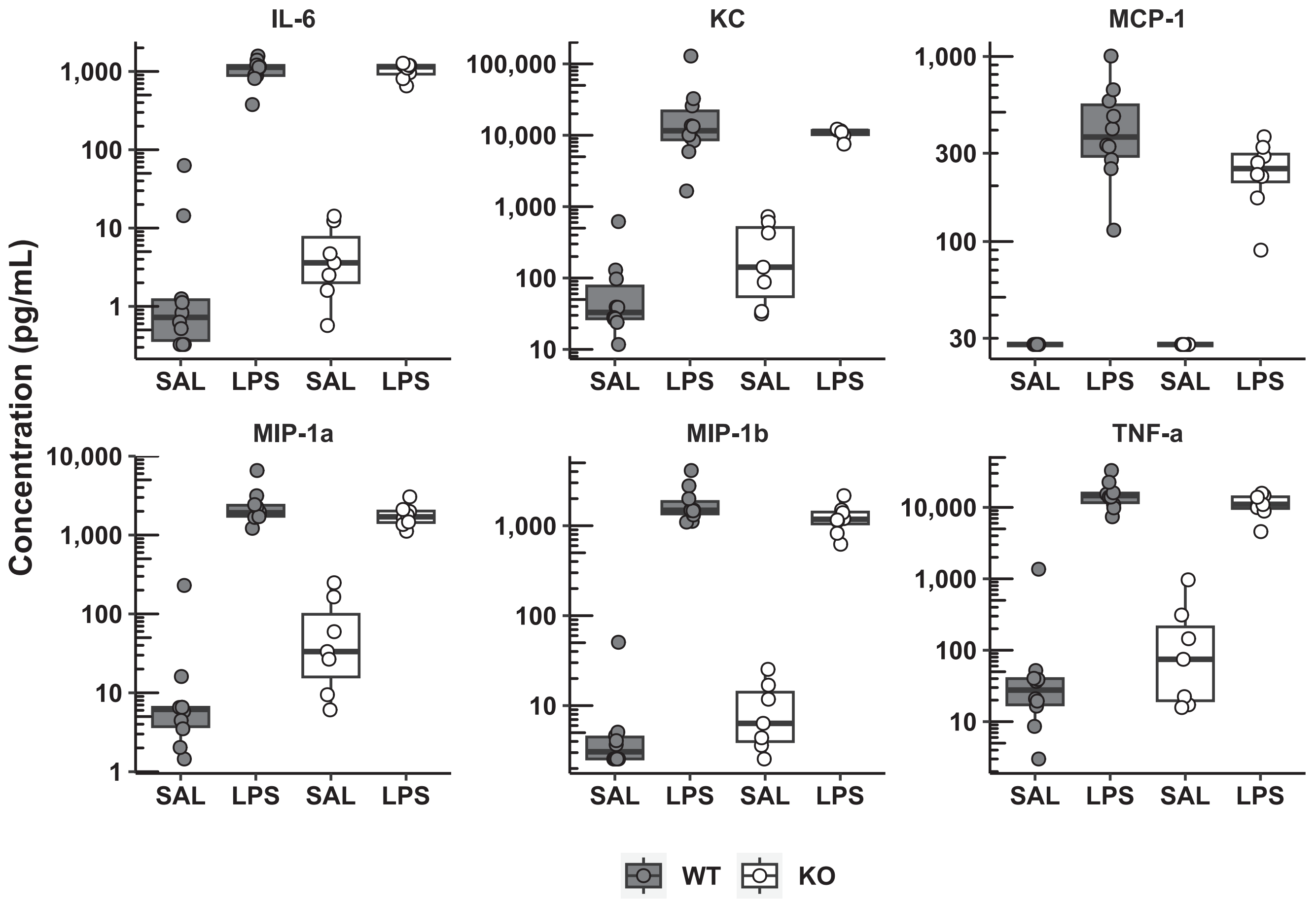


**Fig. S10 Cytokine levels in bronchoalveolar lavage fluid (BALF) in mice following LPS exposure.** The increased degrees of cytokines (IL-6, KC, MCP-1, MIP-1a, MIP-1b, and TNF) following LPS (vs. saline) were not different in the *Adam19* KO and WT mice. SAL=Saline. WT: n=10 (SAL), 10 (LPS); KO: n=7 (SAL), 8 (LPS). Y axes are presented in log scale. All p values for differences by genotypes of cytokine changes following LPS (vs. saline) were greater than 0.05.
